# Heterologous viral protein interactions within licensed seasonal influenza virus vaccines

**DOI:** 10.1038/s41541-019-0153-1

**Published:** 2020-01-10

**Authors:** Marina Koroleva, Frances Batarse, Savannah Moritzky, Carole Henry, Francisco Chaves, Patrick Wilson, Florian Krammer, Katherine Richards, Andrea J. Sant

**Affiliations:** 1 David H. Smith Center for Vaccine Biology and Immunology, Department of Microbiology and Immunology, University of Rochester Medical Center, Rochester, NY USA; 2 Department of Medicine, Section of Rheumatology, The University of Chicago, Chicago, IL USA; 3 Department of Microbiology, Icahn School of Medicine at Mount Sinai, New York, NY USA

**Keywords:** Protein vaccines, Cellular immunity, Humoral immunity

## Abstract

Currently, licensed influenza virus vaccines are designed and tested only for their ability to elicit hemagglutinin (HA)-reactive, neutralizing antibodies. Despite this, the purification process in vaccine manufacturing often does not completely remove other virion components. In the studies reported here, we have examined the viral protein composition of a panel of licensed vaccines from different manufacturers and licensed in different years. Using western blotting, we found that, beyond HA proteins, there are detectable quantities of neuraminidase (NA), nucleoprotein (NP), and matrix proteins (M1) from both influenza A and influenza B viruses in the vaccines but that the composition differed by source and method of vaccine preparation. We also found that disparities in viral protein composition were associated with distinct patterns of elicited antibody specificities. Strikingly, our studies also revealed that many viral proteins contained in the vaccine form heterologous complexes. When H1 proteins were isolated by immunoprecipitation, NA (N1), M1 (M1-A), H3, and HA-B proteins were co-isolated with the H1. Further biochemical studies suggest that these interactions persist for at least 4 h at 37 °C and that the membrane/intracytoplasmic domains in the intact HA proteins are important for the intermolecular interactions detected. These studies indicate that, if such interactions persist after vaccines reach the draining lymph node, both dendritic cells and HA-specific B cells may take up multiple viral proteins simultaneously. Whether these interactions are beneficial or harmful to the developing immune response will depend on the functional potential of the elicited virus-specific CD4 T cells.

## Influenza vaccines: Multiple viral proteins in licensed vaccines

Licensed influenza virus vaccines are evaluated for their ability to elicit neutralizing antibodies specific for hemagglutinin (HA), but the manufacturing process does not completely exclude other virion components from the formulations. Andrea Sant and colleagues now report the presence of several viral proteins, such as M1, NA, H3, and HA-B, in licensed formulations from different manufacturers and spanning stocks from several years. These viral proteins form heterologous complexes, and immunization of mice with some of the formulations analyzed elicited antibody responses specific to these viral proteins. These findings reveal heterogeneity across licensed influenza virus vaccine formulations, potentially due to variations in production processes, and raise the possibility that the presence of these additional viral protein complexes could influence the elicited immune responses following immunization, particularly in the context of multivalent strategies involving mixing of different formulations.

## Introduction

CD4 T cell help is essential for production of high affinity antibodies in response to vaccination (reviewed in refs ^1–3^). The nature and complexity of the immunogen within vaccines will therefore affect the specificity and abundance of CD4 T cell responses and thus potentially the magnitude and quality of the elicited antibody. Licensed inactivated influenza virus vaccines (IIVs) are primarily designed to elicit high-affinity antibodies to hemagglutinin (HA) that can neutralize influenza viruses, thus providing sterilizing immunity to future infection. However, some biochemical studies have suggested that beyond HA, IIV can contain additional influenza viral proteins such as neuraminidase (NA) (reviewed in refs ^4–6^). Recent biochemistry studies have shown that NA in licensed influenza vaccines can now be quantified by stable isotope dimethyl labeling in conjunction with mass spectrometry.^7^ Published studies have also shown that inactivated vaccines can contain other viral components such as matrix protein (M1) and nucleoprotein (NP) and can elicit both CD4 T cell and antibody responses to inactivated influenza vaccination.^8–12^ These studies have suggested the potential of these induced specificities to provide broadly protective immunity to influenza. Our own studies of CD4 T cell responses to licensed vaccines in mice and humans, quantifying virus-specific CD4 T cell reactivity, indicate that in addition to HA and NA, expansion of CD4 T cells specific for NP and M1 occurs.^11,13,14^ The impact of these additional influenza virus proteins in IIV and the responses to them on human immunity to the virus are unknown at this time but can easily be envisioned to have functional consequences in both the B cell and CD4 T cell compartments.

IIV preparation for IIV is completed through several distinct steps that vary somewhat among different manufacturers. For all licensed seasonal IIVs, virus strains are chosen based on the prevalence of circulating viruses identified in the previous season. Twice a year, once for the Northern hemisphere and once for the Southern hemisphere, vaccine formulations are re-evaluated by the World Health Organization, and if needed, new annual recommendations are made.^15,16^ Seed viruses for these individual strains (H1N1, H3N2, and influenza B virus) are constructed and then expanded in either embryonated eggs or, most recently, mammalian cells (reviewed in refs ^17,18^). The virions are then inactivated, purified, and disrupted, typically with detergents. Subsequent purification steps are largely proprietary for each vaccine manufacturer but typically include high-speed centrifugation and may additionally include column chromatography approaches. Supplementary Table 1 represents a summary of the information provided by different manufacturers for the vaccines used in this study, as provided on the product data sheets. Inactivated IIVs are standardized based on HA content, as determined by single radial immunodiffusion assays, which guarantees a minimal quantity per dose. In the final stages of formulation, H1N1, H3N2, and influenza B components are combined at the required minimal concentrations of HA protein. In typical licensed vaccines, the dose is 15 µg of each HA protein (H1, H3, HA-B), while the vaccine specifically designed for the elderly^19^ is administered at a dose of 60 µg for each HA protein. Most recently, there has been inclusion of two distinct circulating strains of influenza B virus.^20,21^ Such quadrivalent inactivated vaccines (QIVs) incorporate two strains of influenza B virus in order to provide protection for both the B/Yamagata/16/88-like and B/Victoria/2/87-like lineages,^20,21^ in addition to H1N1 and H3N2. IIVs are further classified into two different categories “split-virion” or “subunit-virion” vaccines. These vaccines differ in their purification methods, and in their final composition, with subunit vaccines generally undergoing additional purification steps designed to enrich for HA and diminish the abundance of other viral components in the final formulated product.^22,23^

In the studies described here, we have sought to gain a more generalized understanding of the composition of licensed IIVs, characterizing viral protein presence and relative abundance among different vaccines, prepared in different years and by different manufactures. With the goal of better understanding the factors that contribute to the adaptive immune responses to influenza vaccination, we have also asked whether the proteins contained in the vaccines physically interact with each other. These studies have shown a surprising degree of complexity in the licensed vaccines, suggesting that they can simultaneously recruit responses by CD4 T cells and B cells of distinct viral protein specificities. We have also discovered that heterologous viral proteins, including M1, NA, H3, and HA-B, form stable complexes with the H1 protein in the licensed influenza vaccines, raising the possibility that they have the potential to be co-delivered to antigen-presenting dendritic cells, macrophages, or influenza-specific B cells.

## Results

### Licensed influenza vaccines are highly complex with regard to viral protein composition

**Fig. 1:**
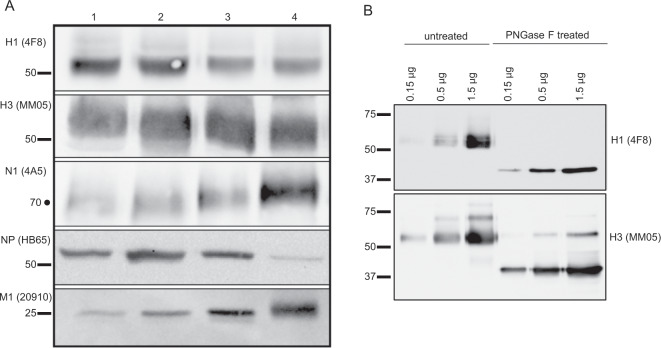
Viral protein composition of licensed seasonal influenza vaccines. In **a**, licensed seasonal influenza vaccines Fluzone 2014–15 (Lane 1), Fluzone 2015–16 (Lane 2), FluLaval 2013–14 (Lane 3), and FluLaval 2015–16 (Lane 4) were surveyed for the presence of H1 (55 kDa), H3 (55 kDa), NA (75 kDa), NP (56 kDa), and M1 (27 kDa) using antibodies listed in Supplementary Table 2 and indicated to the left. Vaccines were prepared to a final concentration of 2.7 µg of total HA per sample for immunoblot, based on manufacturer-reported hemagglutinin quantity. The proteins within the vaccines were fractionated by SDS-PAGE gel and then analyzed by western blot as described. In **b**, Fluzone proteins in the 2017–18 vaccine were treated with the enzyme PNGase-F to remove any N-linked glycans present on the HA proteins and then probed for H1 and H3. Untreated vaccines, applied at varying concentrations of HA, are shown on the left and the PNGase-treated vaccines are shown on the right. Molecular weight markers are indicated. A dot represents a molecular weight extrapolated from the mobility of the molecular weight markers.

We first performed a survey of four different split virion licensed IIV (Fluzone 2014–2015, 2015–2016 and FluLaval 2013–2014, 2015–2016) prepared from embryonated chicken eggs in order to characterize their viral protein composition. Aliquots, normalized to HA content reported by the manufacturer, were applied to sodium dodecyl sulfate (SDS)-10% polyacrylamide gel electrophoresis (PAGE) gels under reducing conditions, separated electrophoretically, and transferred to nitrocellulose. Blots were probed for H1, H3, N1, NP, and M1, as indicated on the left of Fig. 1a, using antibodies indicated in Supplementary Table 2. These experiments revealed that, across two consecutive influenza seasons (2014–2015 and 2015–2016), all of the split virion vaccines had readily detectable NA, M1-A, and NP in addition to H1 and H3 proteins. Fluzone had similar concentrations of H1 in both of the years tested, which was somewhat higher than that detected in FluLaval. To compare the abundance of the influenza virus proteins within these vaccines, as well as an estimate of reproducibility across independent experiments, densitometry analyses were performed (Supplementary Fig. 1). Examination of these data suggest that the assays are reproducible and that while HA content can differ by twofold among vaccines as estimated by western blotting, NA, NP, and M1 content can vary as much as 3–4-fold, depending on the year of production and the manufacturer. These data argue that the composition of viral proteins in split vaccines is complex, and in addition to the HA proteins derived from each virus strain, the administered vaccine contains NA, NP, and M1, present in variable quantities per dose, depending on the year and vaccine manufacturer. We noted that, in Fig. 1, there was considerable heterogeneity in SDS-PAGE gel mobility in the HA proteins from each of the manufacturers, which was not detectable with M1 and NP proteins. We speculated that the complexity of these patterns might be due to both variability and processing of HA0 to both HA1 and HA2, as noted previously,^24^ as well as microheterogeneity in the glycosylation patterns of the HA proteins.^25^ To examine this latter possibility, the licensed split vaccine Fluzone was treated with PNGase F and we compared the SDS-gel mobility of the treated sample relative to the control. Figure 1b shows that both H1 and H3 are reduced in apparent molecular weight by the treatment, and with this loss in molecular weight, their mobility on SDS-PAGE becomes very focused, consistent with the interpretation that the HA proteins prepared in vaccines have variable N-linked carbohydrate side chains.

**Fig. 2:**
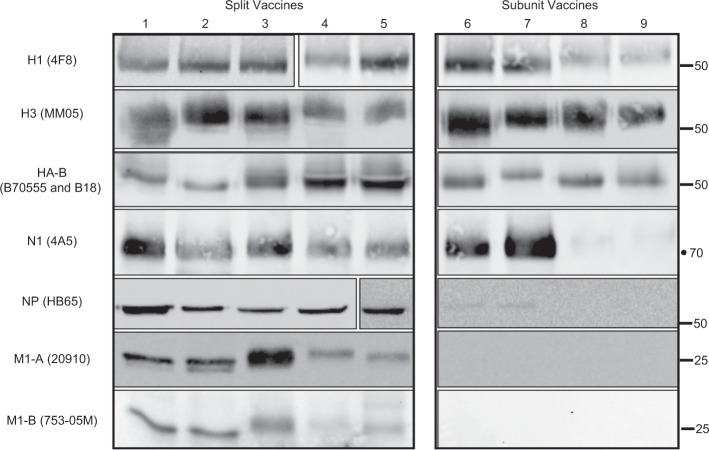
Viral protein composition of split and subunit vaccines. Licensed seasonal influenza vaccines Fluzone 2015–16 (Lane 1), FluLaval 2013–14 (Lane 2), FluLaval 2015–16 (Lane 3), Fluarix 2015–16 (Lane 4), Fluarix 2016–17 (Lane 5), Fluvirin 2015–16 (Lane 6), Fluvirin 2016–17 (Lane 7), Flucelvax 2015–16 (Lane 8), and Flucelvax 2016–17 (Lane 9) were surveyed for the presence of H1, H3, HA-B, NA, NP, M1-A, and M1-B, as indicated to the left, using antibodies listed in Supplementary Table 2. Split virus vaccines are shown in the left panel and subunit vaccines in the right panel. Vaccines were prepared to a final concentration of 2.7 µg of total HA per lane for immunoblotting, fractionated by SDS-PAGE gel, and then analyzed by western blot, as described.

We then extended our studies of viral protein composition and included additional split vaccines and two subunit vaccines. “Subunit” influenza vaccines undergo additional purification steps prior to final formulation intended to highly enrich for the HA protein.^22^ We also modified our experiments to include detection of proteins from influenza B viruses, HA-B and M1-B, using antibodies that are able to recognize the proteins from the two different lineages of influenza B virus (B/Yamagata/16/88-like and B/Victoria/2/87-like) that are included either alone (in trivalent inactivated vaccine (TIV)) or together (in QIV). The results of these experiments are shown in Fig. 2. The vaccines analyzed include, from left to right, split vaccines Fluzone 2015–16, FluLaval 2013–14, FluLaval 2015–16, Fluarix 2015–16, and Fluarix 2016–17, and subunit vaccines Fluvirin 2015–16 and Fluvirin 2016–17, all produced in eggs. We also included vaccines prepared in mammalian cell culture (Flucelvax)^26^ from two seasons (2015–16 and 2016–17) shown at the far right. Western blot analyses confirm that, in addition to H1, H3, and HA-B detected in each of the tested IIVs, the split vaccines contain NA, NP, M1-A, and M1-B, while the subunit vaccines tested here have very little detectable internal virion proteins NP, M1-A, and M1-B, consistent with the goal of producing more highly purified HA proteins for vaccination. In addition, although all of the split vaccines and the egg-derived subunit vaccine Fluvirin contain readily detectable N1 proteins, the Flucelvax subunit vaccine, prepared from cultured Madin Darby Canine Kidney cells, contains very little N1, as detected by the monoclonal antibody (mAb) 4A5, which has been shown to react broadly to many different N1 proteins.^6^ These findings are in agreement with earlier studies showing the variable content of NA in several licensed vaccines.^6^

### The quantity of non-HA proteins within licensed vaccines correlates with the elicitation of an immune response in vivo

**Fig. 3:**
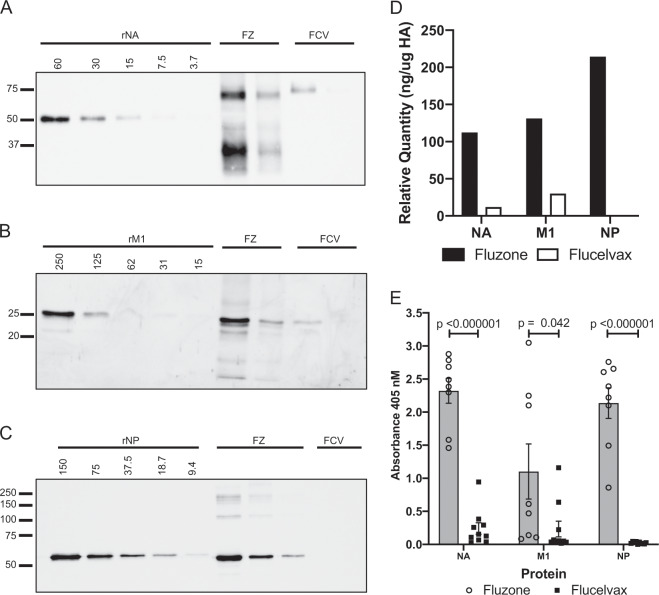
Quantification and immunogenicity of NA, M1, and NP in influenza vaccines. In **a**–**c**, influenza proteins NA, M1, and NP were quantified using western blotting as a semi-quantitative method, using recombinant proteins shown in the left lanes, at known concentrations. In panel **a**, recombinant NA protein was applied in twofold dilutions starting at 60 ng. Fluzone (FZ) 14–15 and Flucelvax (FCV) 15–16 were applied at 1.8 or 0.6 µg HA, respectively. In panel **b**, recombinant M1 protein was applied in twofold dilutions starting at 250 ng. Fluzone (FZ) 14–15 and Flucelvax (FCV) 15–16 were applied at 1.8 or 0.6 µg HA, respectively. In panel **c**, recombinant NP protein was applied in twofold dilutions starting at 150 ng, Fluzone (FZ) 14–15 was applied at 0.6, 0.2, and 0.07 µg HA, and Flucelvax (FCV) 15–16 was applied at 1.8 µg HA. Standard curves were generated based on the densitometry values of the recombinant protein titrations. **d** represents the quantifications of the viral proteins in Fluzone (filled) and Flucelvax (open) from the blots shown in **a**–**c**. In **e**, serum antibodies from mice vaccinated with Fluzone (filled) or Flucelvax (open) were tested for reactivity to recombinant NA, M1, and NP, from left to right. IgG antibodies were quantified by ELISA assays at a fixed serum dilution. Shown is the average of 8–10 individual animals, with individuals indicated. Error bars represent the standard error of the mean and Student’s *t* test was used to calculate *p* values.

To gain a semi-quantitative estimate of the abundance of NA, NP, and M1-A in the vaccines, we tested two vaccine types that exhibited striking disparities in viral protein composition (Fluzone and Flucelvax), as shown in Fig. 2, lane 1 and lanes 8–9, respectively. An aliquot of each vaccine was applied directly to an SDS-PAGE gel, and in parallel, recombinant M1, NP, and NA proteins were applied in graded quantities to the same gel, fractionated by electrophoresis, and transferred to nitrocellulose, as before. Blots were probed for the viral proteins, and from the quantified signal obtained by chemiluminescence, we were able to estimate the protein concentration of M1, NP, and NA in the vaccines and compare them between the two vaccines. The results of these experiments are shown in Fig. 3, where the NA, M1, and NP proteins were quantified by using standard curves determined from the titration of recombinant protein (Fig. 3a–e, respectively). Fig. 3d illustrates the approximate quantity of each of these proteins in the vaccines, revealing that the Fluzone vaccine tested here contains approximately tenfold and fivefold more NA and M1, respectively, than Flucelvax, which additionally contained no detectable NP.

To evaluate whether the abundance of M1, NP, and N1 in the split vaccine Fluzone and the relative paucity of these proteins in subunit vaccine Flucelvax led to different patterns of immune responses, separate cohorts of mice were vaccinated subcutaneously in the footpad with either Fluzone or Flucelvax vaccine in alum. Serum samples collected at day 13 postvaccination were tested for the presence of antibodies to NA, M1, and NP proteins by enzyme-linked immunosorbent assay (ELISA) assays. Shown in Fig. 3e are the results of these assays. Fluzone elicited readily detectable antibodies to M1, NP, and N1. However, antibody responses induced by Flucelvax to these non-HA proteins were almost undetectable. Elicited HA-specific antibodies by these two vaccines were equivalent in these studies (Supplementary Fig. 2). These results suggest that the varying composition of licensed influenza vaccines can dramatically affect the breadth of the induced antibody responses to vaccination.

### Vaccines contain heterologous inter-protein complexes, including cross-HA strain complexes

How the viral proteins administered in a vaccine are presented to lymphocytes may have a significant impact on CD4 T cell priming and generation of a high affinity neutralizing HA-specific antibody response. Because of the complex viral protein composition in the IIVs tested, we sought to investigate whether the viral proteins within the vaccines displayed any intermolecular viral protein interactions. We speculated that, because the viral surface membrane proteins are transmembrane proteins, they might co-isolate together. Detergents are commonly used to disrupt the influenza virions during the preparation of the vaccine but the subsequent purification steps are thought to remove most of the detergent prior to formulation, a logical step for a clinically administered human vaccine. However, we considered the possibility that transmembrane proteins such as HA and NA, having intact hydrophobic domains in their membrane-spanning regions, combined with removal of the detergent during formulation, may lead to aggregation of these viral surface proteins via hydrophobic interactions of their transmembrane domains.

To analyze interactions among IIV proteins, co-immunoprecipitation (co-IP) assays on split IIVs were performed on samples as supplied by the manufacturer, typically in saline solution. We sought to evaluate proteins associated with the H1 influenza virus protein as a prototype. Protein G Sepharose (PGS) was used to prepare an immunoadsorbent with either a human antibody specific to H1 or a control antibody. The immunoadsorbents were incubated with an aliquot of the vaccine overnight to allow isolation of the H1 proteins. H1 proteins present within the vaccine would thus have the opportunity to bind the immunoadsorbent, and with it, any associated proteins within the vaccine would be co-isolated. After extensive washing in phosphate-buffered saline (PBS), material remaining bound to the immunoadsorbents was eluted in SDS sample buffer and applied to SDS-PAGE, followed by western blot analysis.

**Fig. 4:**
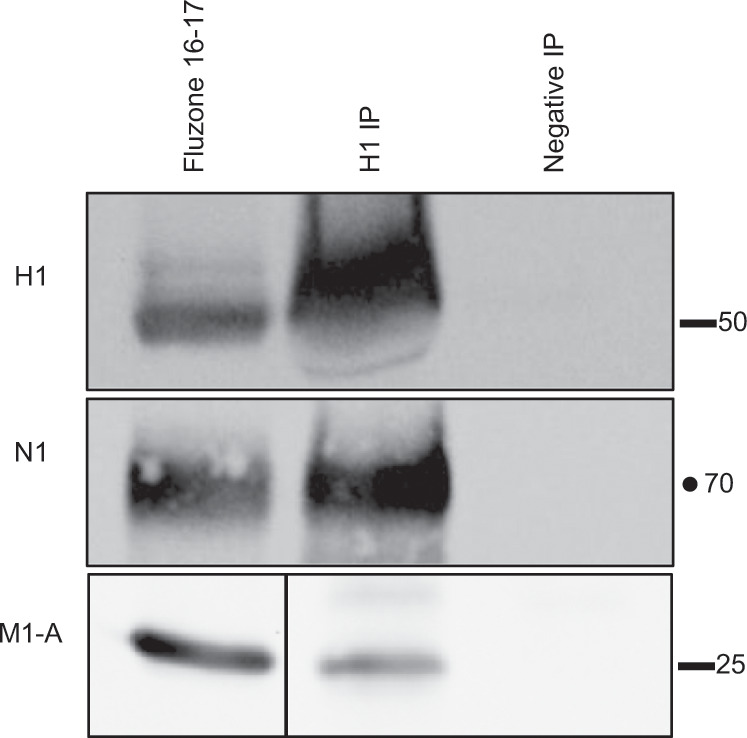
N1 and M1 in licensed vaccine co-isolate with H1. Co-immunoprecipitation assays were used to detect protein–protein interactions in the split vaccine Fluzone 2016–17. Protein G Sepharose was used to prepare an immunoadsorbent with either a human antibody specific to H1 (H1 IP) or control antibody (Negative IP), the immunoadsorbents were mixed with an aliquot of the vaccine overnight, and after washing, bound material was eluted in SDS-sample buffer and applied to SDS-PAGE, followed by western blots probed for N1 and M1-A. In each experiment, an aliquot of the total vaccine was applied to the gel and is shown in the left most lane.

**Fig. 5:**
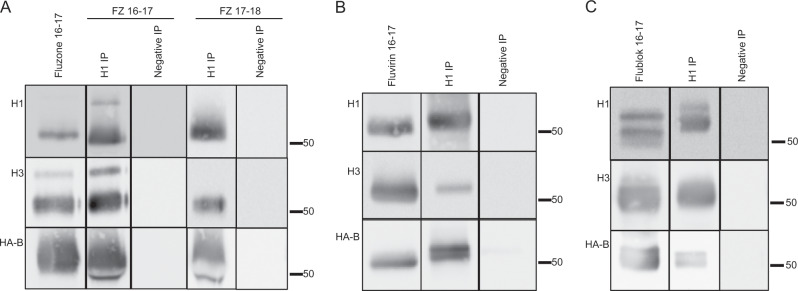
Heterologous hemagglutinin proteins co-isolate with H1 in influenza vaccines. Co-immunoprecipitation assays were used to detect interactions between the hemagglutinin proteins in Fluzone 2016–17 or 17–18 (**a**, left and right, respectively), Fluvirin 16–17 (**b**), and Flublok 16–17 (**c**). Protein G Sepharose was used to prepare an immunoadsorbent with either a human antibody specific to H1 (Lane 2 and 4, H1 IP) or control antibody (Lane 3 and 5, Negative IP). The immunoadsorbents were prepared by incubating an aliquot of the vaccine overnight to allow isolation of the H1 proteins and other HA proteins associated with H1. After extensive washing in PBS, material remaining bound to the immunoadsorbents was eluted in SDS sample buffer and applied to SDS-PAGE, followed by western blot analysis. In each experiment, an aliquot of the total vaccine was applied to the gel (Lane 1).

Figure 4 shows the results of these co-IP experiments using the split vaccine Fluzone 2016–2017 for detection of NA (N1) or M1 (M1-A) in association with the H1 protein. These studies revealed that both N1 and M1 were readily detected within the H1-specific immunoprecipitate, indicating that, within the vaccine, H1, N1, and M1 interact with each other. We then extended our studies of protein–protein interactions to ask whether the different HA proteins, each the main targets of vaccination within licensed vaccines (H1, H3, and HA-B), are capable of forming heterosubtypic complexes. As before, aliquots of the vaccines were incubated with immunoadsorbents prepared with H1-specific or control antibodies. Isolated proteins were fractionated by SDS-PAGE and analyzed by western blots that were probed with antibodies specific for H3 or HA-B. Figure 5 depicts the results of these experiments with split vaccines Fluzone 2016–2017 and Fluzone 2017–2018 in Fig. 5a and subunit vaccine Fluvirin 2016–2017 in Fig. 5b. It is clear that different strain origin HA proteins (H3 and HA-B) are associated with sufficient affinity with H1 within the vaccine to be co-isolated with the H1 HA protein by IP.

Recently, licensed vaccines have been developed that are derived from insect cells infected with baculovirus vectors encoding H1, H3, or HA-B. These vaccines (Flublok) are prepared by detergent extraction of intact HA proteins from the transfected cells and are then enriched by gel filtration and column chromatography.^27^ As with split and subvirion vaccines, after preparation of the vaccine components from each influenza virus strain, the components are combined to yield either a trivalent or quadrivalent vaccine, depending on the year of production. Because the HA proteins expressed in insect cells, like those prepared from isolated influenza virions, are extracted in an intact form from the infected cells, their membrane and intracytoplasmic segments are intact. We speculated that the HA proteins within these multivalent vaccines might also interact, perhaps through their hydrophobic domains. To test this, the Flublok vaccine was incubated with the H1 immunoadsorbent and the isolated material was analyzed by western blotting. Figure 5c shows that H3 was readily detectable in the H1-specific immunoprecipitate, demonstrating that the interactions between H1 and H3 proteins upon formulation of the split and subunit vaccines do not require other virion proteins. Figure 5c also shows that detection of HA-B was more modest with Flublok and generally less pronounced than in the egg-based vaccines.

Collectively, these co-IP studies lead us to the unexpected conclusion that not only do different viral proteins within a given IIV component (H1-N1, H1-M1) associate with each other after detergent extraction and purification but that proteins from the different components also interact after the subtype-specific vaccine preparations are combined into the vaccine formulation. These co-IP data suggest the formation of mixed viral protein complexes in the format in which the licensed vaccines are administered. In the split and subunit Fluzone and Fluvirin vaccines, respectively, H1 readily co-isolates H3 and HA-B, while in Flublok, the most stable interactions appear to be detected between H1 and H3.

### The heterologous interactions among influenza viral proteins are not detectably altered with vaccine storage and are stable at 37 °C

**Fig. 6:**
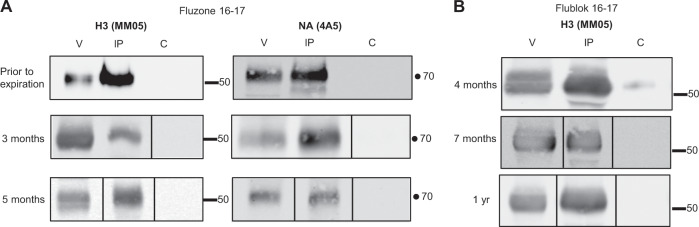
Interaction of heterologous hemagglutinin proteins in influenza vaccines are unchanged over time. Co-immunoprecipitation assays were used to detect interactions between proteins in Fluzone 2016–17 (**a**) and Flublok 16–17 (**b**) from prior to vaccine expiration to a year past expiration. Protein G Sepharose was used to prepare an immunoadsorbent with either a human antibody specific to H1 (Lane 2, “IP”) or control antibody (Lane 3, “C”). The immunoadsorbents were prepared by incubating an aliquot of the vaccine overnight to allow isolation of the H1 proteins and other proteins associated with H1. After extensive washing in PBS, material remaining bound to the immunoadsorbents was eluted in SDS sample buffer and applied to SDS-PAGE, followed by western blot analysis. The antibody specificities, H3 or N1, are indicated above the image. In each experiment, an aliquot of the total vaccine was applied to the gel (Lane 1, “V”).

Several questions can be raised about the cross-viral HA interactions observed within the licensed vaccines. First, because we have sampled vaccines across different manufacturing years, one can ask whether these interactions among the HA proteins of different influenza strain origins change upon vaccine storage and are detectable with in-season vaccines, prior to their expiration. Figure 6 shows that no time-dependent changes in cross-HA protein interactions were detectable across up to 12 months of storage, for both split Fluzone vaccine (Fig. 6a) and recombinant HA protein vaccine Flublok (Fig. 6b) where replicates spanning a long time frame were available. In addition, additional vaccines tested (Figs 4 and 5) were examined prior to expiration, and for these, the cross-HA interactions are also readily detectable.

**Fig. 7:**
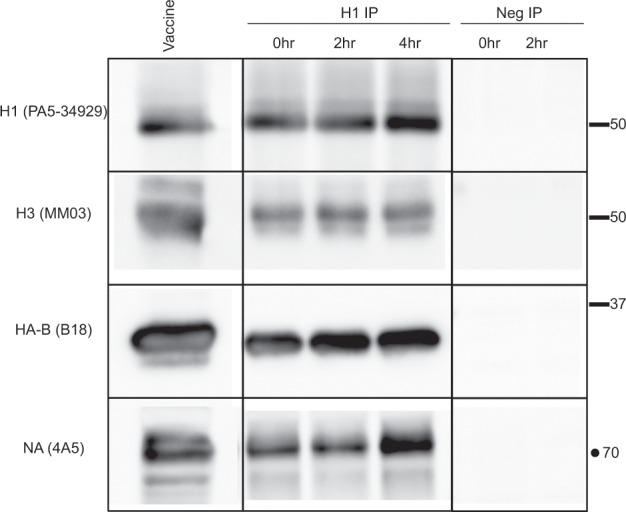
Protein–protein interactions in the seasonal influenza vaccine persist at 37 °C. Co-immunoprecipitation assays were used to detect interactions between the hemaggluttinin proteins in Fluzone 2018–19 under physiological temperature conditions. Protein G Sepharose was used to prepare an immunoadsorbent with either a human antibody specific to H1 or control antibody. Vaccine was incubated at 37 °C for 2 or 4 h in the presence of 1% BSA, and immunoadsorbents were then prepared by incubating the vaccine samples overnight to allow isolation of the H1. After extensive washing in PBS, material remaining bound to the immunoadsorbents was eluted in SDS sample buffer and the proteins isolated were analyzed by western blotting with the antibody specificities indicated on the left of each panel. In each experiment, a small amount of vaccine was applied to the gel (Lane 1).

Second, it is interesting to consider whether the heterologous protein interactions detected in vaccines could persist until the vaccine reaches the draining lymph node after vaccination. To examine this question, vaccines were incubated in separate aliquots at 37 °C for 2–4 h, with one aliquot stored on ice. Immunoprecipitates were then prepared with either control or H1 antibodies, washed as before, applied to a SDS-PAGE, and analyzed by western blotting. The results of these studies, shown in Fig. 7, indicate that the vaccines are stable for at least 4 h at 37 °C, potentially allowing them to drain as complexes together to the lymph node after vaccination.^28^

### Regions in the membrane proximal domains contribute to cross-HA interactions

**Fig. 8:**
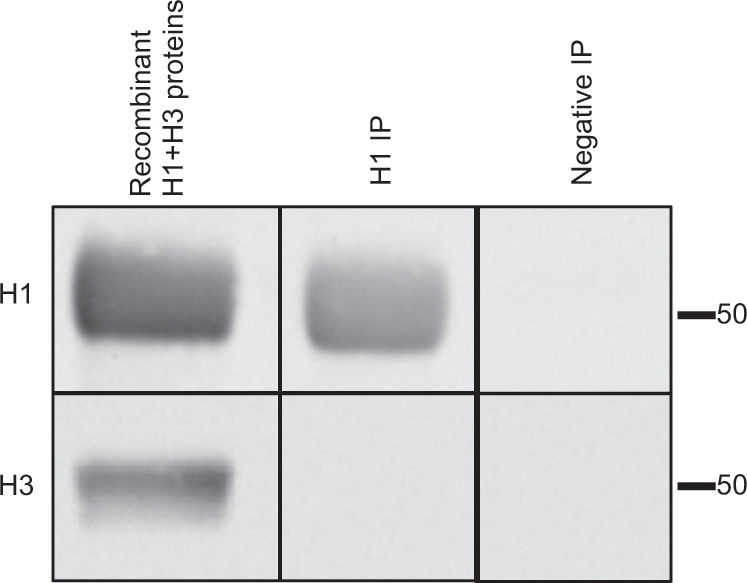
The native transmembrane domain is needed for interactions among HA proteins. H1 A/California/04/09 (H1) and A/Perth/16/09 (H3) soluble recombinant proteins lacking transmembrane domains were combined and tested to determine whether cross-HA interactions are formed. In the left lane, H1 and H3 were mixed just prior to addition of SDS sample buffer and application of the gel. To test for H1–H3 interactions, an aliquot of recombinant H1 and H3 proteins were mixed and incubated overnight. Immunoadsorbents with either a human antibody specific to H1 (H1 IP) or a control antibody (Negative IP) and then were used to collect H1 proteins and any associated H3 in the mixed sample. Immunoprecipitated samples were eluted in SDS sample buffer, fractionated by SDS-PAGE, and the proteins isolated were analyzed by western blotting with the antibody specificities indicated on the left.

We sought to gain insight into the mechanisms that contribute to the protein–protein interactions observed within the vaccines. We hypothesized that, after removal of detergent by the vaccine manufacturer, proteins with hydrophobic domains such as HA and NA might tend to aggregate together in small detergent/lipid micelles, and then upon mixing the individual vaccine components into trivalent or quadrivalent vaccines at high concentration, the components would intermix and exchange with each other. One prediction of this model is that recombinant soluble HA proteins with truncated transmembrane domains might not exhibit these interactions. To address this question, we next examined recombinant influenza H1 and H3 virus HA proteins produced as soluble proteins from the supernatant, using a strategy described by Nabel and co-workers.^29^ Here the extracellular domain of HA is fused to a trimerization domain, which is linked to a hexahistidine tag used for purification of the HA using Ni+ column chromatography. H1 (A/California/04/09) and H3 (A/Perth/16/09) proteins made using these constructs were mixed and incubated overnight. To account for any non-specific protein–protein interactions, we also included bovine serum albumin (BSA) in the samples. Anti-H1 immunoadsorbents were then prepared as before, and the eluates were fractionated by SDS-PAGE and analyzed by western blotting. The results of these experiments are shown in Fig. 8. Lane 1 shows that both soluble, recombinant H1 and H3 applied directly to the gel are readily detectable in the mixed sample applied and probed by western blotting (Fig. 8 left lane). However, after mixing and co-incubation, H3 did not gain any detectable interactions with H1 (middle lane). These results demonstrate both the specificity of the H1 antibody used for IP in all of the studies described here and indicate that the interactions among the alternative subtypes of HA in vaccines requires intact transmembrane and intracytoplasmic domains, as exists in Flublok and the HA proteins in vaccines prepared from influenza virions.

## Discussion

Here we have undertaken a study to better evaluate the viral protein composition and molecular interactions in licensed IIVs. We first surveyed different licensed IIVs to assess their viral protein composition and found that, in addition to H1, H3, and HA-B, split vaccines from many manufacturers and in different years of production contain variable but readily detectable quantities of N1, NP, and M1. We then assessed whether any of the proteins within the vaccines interact with each other with sufficient stability to be co-isolated together. We found that M1 and N1 were isolated in association with H1 and that HA molecules isolated from different virus strains (H1N1, H3N2, and influenza B virus) also formed stable complexes with each other. These results suggest that, after the viral proteins are extracted from the intact and inactivated virions during the “splitting” process, purified, and then combined into a single vaccine formulation, the distinct viral proteins mix and associate with each other. The mechanism that underlies the formation of the complexes or the physical nature of the complexes is unknown at this time. They may either be present in large mixed aggregates or smaller mixed protein complexes. We speculate that, after the process of detergent solubilization and removal of detergent, typically via centrifugation, dialysis, or through use of matrices that selectively bind the detergent,^17,18^ the concentration of the detergent is sufficiently reduced to leave only small micellar complexes, with the short hydrophobic transmembrane segments on the HA and NA proteins binding residual detergent. Upon mixing the three or four vaccines into the combined format (in TIV or QIV, respectively) at the concentrations needed for vaccination, the different HA proteins, as well as NA and M1, may mix into different combinations of complexes. Consistent with this view that the transmembrane segments of HA contribute to the cross-protein interactions, we found that, while H1 and H3 within Flublok, which is composed exclusively of intact HA expressed in transfected insect cells and extracted by detergent, do exhibit stable interactions between H1 and H3, recombinant H1 and H3 proteins in which the transmembrane and intracytoplasmic domains are eliminated, allowing secretion of the HA molecules and purified without the need for detergents, do not display any association. These results suggest that sequences or characteristics of the transmembrane/cytoplasmic domains contribute to the cross-subtype interactions. Possibilities for these interactions include non-covalent association of hydrophobic domains of the transmembrane domains that could aggregate when detergent/lipids are reduced or oxidation of cysteines within the transmembrane and intracytoplasmic domains of HA that have been shown to form covalent bonds during processing and purification of the purified protein HA in Flublok or during extended storage at room temperature (RT).^30,31^

Parallel sampling of many vaccine lots in this comparative study necessitated analysis of some vaccines past their expiration date. This might raise the question of whether the proteins we have quantified and the interactions we have studied here have been modified during storage. Our existing data suggest that, although some variability exists in detection of the viral proteins in the vaccines made in separate vaccine seasons, this does not appear to be dependent on the time of storage, as our replicate experiments have spanned more than a year with no apparent time-dependent changes in composition or protein–protein interactions. In addition, some of our experiments have involved vaccines prior to their expiration date and our results have not varied from what we have observed in the older vaccines and that are presented here.

It is interesting to consider the implications of our findings on human vaccine responses. First, the complex viral composition in most split vaccines suggest that many distinct viral epitope specificities of CD4 T cells could be elicited after vaccination. In sampling peripheral blood from many healthy adults, our laboratory has found that circulating influenza virus-specific CD4 T cells display broad viral antigen specificity and include CD4 T cells reactive with epitopes from NP, NA, and M1 in addition to HA.^32^ There is particularly robust reactivity toward epitopes from the internal virion proteins NP and M1, in agreement with the studies of other groups.^8–12,33–35^ Circulating memory CD4 T cells specific for these different viral proteins may have become accumulated at least in part via vaccination and have the potential to respond to each subsequent IIV. In fact, in two separate published studies from our laboratory using monovalent inactivated IIVs, we have found vaccine-induced expansion of CD4 T cells specific for influenza M1 and NP epitopes in addition to HA-derived epitopes^13,14^ in agreement with the studies by other groups.^8–12^ The consequences of the responses to influenza viral epitopes other than HA is not known at this time but is important to consider. The response to annual vaccination with split vaccines such as Fluzone or Fluvirin could regularly and repeatedly boost CD4 T cells specific for internal virion proteins, such as NP and M1. This breadth in the response could be beneficial to the host in that these viral proteins are highly genetically conserved and thus have the potential to provide broad protection toward many influenza virus strains. However, it is also important to consider the possibility that repeated boosting of influenza virus-specific CD4 T cells by annual vaccination with IIV may alter the phenotype or gene expression program of the CD4 T cells, as some studies on CD8 T cells in mouse models of infection have shown.^36^ The phenotype of CD4 T cells specific for different viral proteins is poorly understood at this time. In a limited analysis of circulating human CD4 T cells specific for HA and NP, our laboratory has found that HA reactive cells had a higher proportion of T follicular helper cells than did NP-reactive cells,^37^ while HA-B-specific cells had a higher frequency of cytotoxic effectors than did H1 or H3^32^ specific cells. The degree of conservation of these different viral proteins and their abundance in vaccines could each play a role in the evolution of the effector function of CD4 T cells in the human host, as could the original priming event (priming vs. infection) and the frequency of infection over the lifetime of the human host.

It is also important to consider the potential functional consequences of mixed viral protein complexes identified here on the B cell response to influenza virus vaccination. We can envision both positive and negative consequences of these interactions. If the complexes persist after the intramuscular administration of vaccine and trafficking to the draining lymph node, some of the distinct viral proteins in the vaccine will be taken up by the same dendritic cells. For the activation of CD4 T cells, these dendritic cells will simultaneously display a viral-derived epitope from diverse viral proteins. This could potentially influence the specificity of the CD4 T cell response. If CD4 T cells specific for the internal virion proteins such as M1 outnumber those specific for HA and NA, as our own studies suggest,^13,14,32^ they could either provide “bystander help” for the more modest responses of naive cells or outcompete the HA- and NA-specific CD4 T cells. Competition might restrict the priming and boosting of some specificities in the CD4 T cell compartment. Also, the more abundant interferon (IFN)-γ-producing CD4 T cells specific for M1 may antagonize elicitation of naive CD4 T cells specific for new HA and NA epitopes. Our own data suggest that abundant IFN-γ can induce suppression of more slowly evolving CD4 T cell priming^38,39^ and studies by others suggest that robust IFN-γ production can lead to induction of regulatory T cells.^40–42^ During B cell recognition of antigen, mediated through capture by surface immunoglobulin, mixed protein complexes could potentially “misdirect” a fraction of the vaccine. For example, if H3- or HA-B-specific B cells internalize the mixed complexes, this could deplete some of the H1 antigen that will be available to H1-specific B cells. The relative frequency of influenza virus-specific B cells, particularly memory cells, for the different viral antigens would likely determine the degree and consequences of this misdirection of antigen. The same effect might occur if circulating influenza-specific antibodies clear the vaccine prior to initiating the response. Mixed viral protein complexes might also potentiate the antibody response to vaccination. If HA-specific B cells take up multiple viral proteins via their immunoglobulin receptor, they will process and present a diverse set of peptides on their cell surface major histocompatibility complex class II molecules. This would allow these HA-specific B cells to recruit additional CD4 T cell help for expansion, isotype switch, and affinity maturation that takes place in the germinal center. Clearly, the net effect of the diverse viral proteins being administered via IIV and the consequences of mixed viral protein complexes will require more complex studies of the elicited CD4 T cells, their functionality, and, if possible, a direct examination of the epitopes displayed by HA-specific B cells during the developing immune response.

## Methods

### Antibodies

Antibodies were obtained from commercial vendors, Biodefense and Emerging Infections Research Resources Respiratory (BEI Resources), or were provided by collaborators. A complete list of antibodies is provided in Supplementary Table 2. The antibodies were tested for their specificity, optimal concentrations, and application performance in ELISA and western blot assays prior to use.

### Recombinant HA protein

The H1 A/New Caledonia/20/99 plasmid construct was originally obtained from Dr. Barney Graham at the US National Institutes of Health Vaccine Research Center^43^ and these constructs have been described in detail.^29,44^ Briefly, the extracellular domain of HA is C-terminally fused to trimeric T4 fibritin trimerization domain featuring a hexahistidine tag at the C-terminus. We have introduced A/California/04/09 or A/Perth/16/09 HA gene segments into these constructs. Constructs were independently transfected into 293Freestyle cells using 293Fectin (Life Technologies), and cells were then cultured for 3–4 days. The supernatant was harvested, concentrated fivefold using tangential flow filtration, and incubated with PBS (pH 7.4) washed with Ni-nitrilotriacedic acid (NTA) coupled Sepharose beads (GE LifeSciences) overnight at 4 °C. The supernatant–bead mixture was passed through a gravity flow column, and the beads were washed with 15 mM imidazole in PBS, followed by elution of the HA with 500 mM Imidazole in PBS. The eluted fraction was concentrated and the HA trimer purified by high-performance liquid chromatography (HPLC) size exclusion chromatography, concentrated, and the buffer exchanged to PBS. The quantity and quality of the isolated HA trimer was assessed by HPLC, SDS-PAGE, protein concentration, and ELISA.

### NA, NP, and M1 recombinant proteins

Recombinant NA (NR-19234) used in the western blotting was obtained from BEIR. The recombinant NA used in this study was generated by using a baculovirus expression system, where the globular head domain of the respective NA was cloned into a baculovirus shuttle vector, containing an N-terminal signal peptide sequence, was followed by a 6× histidine purification tag, a VASP (vasodilator-stimulated phosphoprotein) tetramerization domain, and a thrombin cleavage site. The baculoviruses were passaged in Sf9 cells to obtain high titers and then used to infect High Five cells for protein expression. The cells were infected for 3 days, shaking at 27 °C. The cell culture supernatant containing the soluble protein was separated from the cells by centrifugation at 4000 × *g* for 10 min. The supernatant was incubated with Ni-NTA agarose beads (QIAGEN) for 3 h, shaking at RT, and the proteins then purified by affinity chromatography using polypropylene columns (QIAGEN). The protein was concentrated and buffer exchanged by using 30 kDa amicon filter units (EMD Millipore) and then stored at −80 °C for further usage. For recombinant M1 and NP proteins, Sf9 cells were infected with a multiplicity of infection of 1 with the respective baculovirus for 5 days at 27 °C. The cells were harvested by centrifugation at 4000 × *g* for 10 min. The supernatant was discarded and the cells were lysed by sonication, followed by a centrifugation for 30 min at 4000 × *g* to remove cell debris. Afterwards, the cleared cell lysate was incubated with Ni-NTA beads shaking at 4 °C for 1 h and the protein was purified as described above and stored at −80 °C for further usage.

### Cells

High Five cells (BTI-TN-5B1–4, *Trichoplusia ni*) were grown in serum-free Express Five media (Gibco) containing 10% L-Glutamine and 1% penicillin/streptomycin antibiotics mix. Sf9 cells (*Spodoptera frugiperda*) adapted from the cell line ATCC CRL-1711 were maintained in *Trichoplusia ni* medium—Fred Hink (TNM-FH, Gemini Bioproducts) supplemented with 1% penicillin/streptomycin antibiotics mix, 1% pluronic F-68 (Sigma-Aldrich), and 10% fetal bovine serum (FBS). In order to passage the baculoviruses, the media was switched to 3% TNM-FH (1% penicillin/streptomycin, 1% pluronic F-68, 3% FBS).

### Vaccine sample preparation

Commercially available vaccines from multiple manufacturers for the 2013–14, 2014–15, 2015–16, 2016–17, 2017–18, and 2018–19 influenza seasons were obtained from the Influenza Vaccine Research Unit at the University of Rochester, who had purchased them from commercial vendors. The trade names, manufacturers, and viral strains used for the vaccine preparation for each vaccine used are listed in Supplementary Table 1. Vaccines were prepared to a final concentration of 2.7 µg of total HA per sample for immunoblot, unless otherwise indicated, based on the manufacturer-reported HA quantity. Laemmli buffer (final concentration 0.06 M Tris-HCl [pH 6.8], 0.4% SDS, 10% glycerol, 0.3% beta-mercaptoethanol, 0.02 mM bromophenol blue) was added to samples. Vaccine-Laemmli buffer samples were incubated for 15 min at RT, followed by heating at 95 °C for 5 min prior to applying to a gel.

### SDS-PAGE and western blotting

After incubation in the SDS sample buffer to denature viral proteins, samples were briefly centrifuged and then applied to an SDS-PAGE gel (10% acrylamide resolving gel, 4% acrylamide stacking gel) run at 120 V for 1–1.5 h using the Bio-Rad Mini Protean II system to allow separation of the vaccine components. Following separation, proteins were transferred to a nitrocellulose membrane (Bio-Rad) at 250 mA for 150 min, after which the membrane was blocked overnight at 4 °C on a rocker using freshly prepared Blotto (5% [w/v] powdered milk (Sigma), 0.1% [w/v] sodium azide (Sigma), 0.05% [v/v] Antifoam A, diluted in Tris-buffered saline-Tween 20 [TBST]). Membranes were incubated with primary antibodies, based on the optimal concentrations determined empirically, overnight at 4 °C or 1 h at RT, with gentle rocking. Blots were washed with copious amounts of TBST and secondary antibody, based on the host source of the primary antibody used, was applied for 1 h at RT. Blots were again washed with copious amounts of TBST and the LumiGLO Peroxidase Chemiluminescent Substrate Kit (SeraCare) and Bio-Rad Chemidoc Imager were used for detection. Multiple exposure times were collected (5 s–3 min). Images were analyzed using the ImageJ software. All blots within a figure panel are from the same experiment and processed in parallel, unless otherwise indicated. Supplementary Figs. 3–5 show uncropped blots.

### PNGase treatment

Influenza vaccine Fluzone 2017–18 (12 μg total HA) was combined with glycoprotein denaturing buffer (NEB) and water, incubated at 100 °C for 10 min, chilled on ice, and centrifuged for 10 s at 15,000 × *g*. The boiled vaccine was then combined with glycoprotein buffer, 10% NP-40, and PNGase F enzyme (NEB) and incubated at 37 °C for 1 h. Following PNGase treatment, the vaccine sample was applied to SDS-PAGE as described above, at varying HA quantities. Following separation, proteins were transferred to nitrocellulose and probed as described above.

### Densitometry quantification

To quantify the concentration of each protein in the IIVs, ImageJ software was used to perform densitometry analysis. Densitometry values were generated, where the specific bands were outlined in squares of equal area and then a value was generated for each individual band intensity that is a percentage of the sum of all the bands outlined.

### Immunoprecipitation

IP assays were performed using the vaccines Fluzone 2016–2017, Fluzone 2017–18, and Fluzone 2018–19 (Sanofi Pasteur), Fluvirin 2016–17 (Novartis), Flublok 2016–17 (Protein Sciences Corp.), or recombinant H1 A/California/04/09 and H3 A/Perth/16/09. For vaccine immunoadsorbents, 3 µg of antibody was bound to 25 µl of packed PGS (GE Healthcare) by incubating at RT for 30 min while rotating. The human-derived anti-H1, clone 3E05, head reactive mAb with no cross reactivity to H3 was used as the capture antibody for H1 HA^45^ and anti-transglutaminase-2 mAb, clone TG2–2D04^46^ was used to account for any non-specific binding in the IP. Immunoadsorbents were washed and then incubated overnight at 4 °C with gentle rotation. Following overnight incubation, PGS was packed by centrifuging at 68 × *g* for 1 min, vaccine was removed, and the beads were washed with PBS 5 times. For temperature sensitivity, vaccine samples were incubated for 2 or 4 h at 37 °C or stored at 4 °C for the duration of the incubation. Following incubation, H1 or control immunoadsorbents were prepared, incubated with the vaccine as before, and washed 5 times with PBS. Immunoprecipitates were eluted by applying Laemmli buffer, mixed gently, incubating at RT for 15 min, and then incubating at 95 °C for 5 min for complete protein dissociation. Supernatants from the immunoadsorbent were applied at 5% and 95% of the total eluate, fractionated by SDS-PAGE, and analyzed by western blotting, as described above.

### Enzyme-linked immunosorbent assay

C3H female mice, 6–8 weeks old, were purchased from Jackson Laboratories. All mice were maintained in the pathogen-free facility at the University of Rochester according to institutional guidelines.

Mice were immunized subcutaneously in the hind footpads with 50 μl of Fluzone 2014–15 formula or Flucelvax 2015–16 emulsified in alum (Invivogen). Influenza vaccine was dialyzed and concentrated, and each mouse was immunized with 3 μg HA. At 13 days postimmunization, serum was collected. NA-, M1-, and NP-specific antibodies in the sera from individual mice were quantified by ELISA assays. Plates (Costar) were coated with 200 ng of purified protein. Wells were rinsed with PBS, incubated with blocking buffer (3% BSA in PBS), and then diluted serum samples (in 0.5% BSA–PBS) were added to the plates and incubated for 2–3 h at RT. The wells were washed and incubated sequentially with 100 μl/well alkaline phosphatase-conjugated goat anti-mouse IgG secondary antibody (SouthernBiotech) and *p*-nitrophenyl phosphate substrate. After washing, absorbance at 405 nm was read.

### Ethics statement

All mice were maintained in a specific-pathogen free facility at the University of Rochester Medical Center according to the institutional guidelines. All animal protocols used in this study adhere to the AAALAC International, the Animal Welfare Act, and the PHS Guide and were approved by the University of Rochester Committee on Animal Resources, Animal Welfare Assurance Number A3291–01. The protocol under which these studies were conducted was originally approved on March 4, 2006 (protocol no 2006-030) and has been reviewed and re-approved every 36 months with the most recent review and approval on January 23, 2018.

### Reporting summary

Further information on research design is available in the Nature Research Reporting Summary linked to this article.

## Supplementary information

Supplementary Materials

Reporting Summary

## Data Availability

The full complement of data accumulated for these studies is available upon request.
