# Supplementary material for: Heterologous viral protein interactions within licensed seasonal influenza virus vaccines
Source: NPJ Vaccines. 2020 Jan 10;5:3. doi: 10.1038/s41541-019-0153-1 (PMC6954117; doi:10.1038/s41541-019-0153-1)
Supplement: Supplementary file 1 — Supplementary Materials [file 41541_2019_153_MOESM1_ESM.pdf]

Name	Vaccine Type	Valency	Manufacturer	H1N1 Strain	H3N2 Strain	Influenza B - Yamagata	Influenza B - Victoria	Inactivation	Method of disruption	Reported purification method
Fluarix 2015-16	Split virus	Trivalent	GlaxoSmithKline Biologicals	A/Christchurch/16/2010 NIB-74XP	A/Switzerland/9715293/2013 NIB-88	B/Phuket/3073/2013	-	Sodium deoxycholate, formaldehyde	Disrupted during concentration and purification	Zonal centrifugation using linear sucrose density gradient solution containing detergent to disrupt the virus
Fluarix 2016-17	Split virus	Quadrivalent	GlaxoSmithKline Biologicals	A/Christchurch/16/2010 NIB-74XP	A/Hong Kong/4801/2014 NYMC X-263B	B/Phuket/3073/2013	B/Brisbane/16/2008	Sodium deoxycholate, formaldehyde	Disrupted during concentration and purification	Zonal centrifugation using linear sucrose density gradient solution containing detergent to disrupt the virus
FluLaval 2013-14	Split virus	Trivalent	ID Biomedical	A/California/07/2009 X-179A	A/Texas/50/2012 NYMC X-223A	B/Massachusetts/02/2012 NYMC BX-51B	-	UV, formaldehyde	Sodium deoxycholate	Purified by centrifugation
FluLaval 2015-16	Split virus	Quadrivalent	ID Biomedical	A/California/07/2009 NYMC X-179A	A/Switzerland/9715293/2013 NIB-88	B/Phuket/3073/2013	B/Brisbane/16/2008	UV, formaldehyde	Sodium deoxycholate	Purified by centrifugation
Fluvirin 2015-16	Subunit	Trivalent	Novartis Vaccines	A/Christchurch/16/2010 NIB-74	A/Switzerland/9715293/2013 NIB-88	B/Phuket/3073/2013	-	Beta-propiolactone	Centrifugation with nonylphenol ethoxylate	Zonal centrifugation
Fluvirin 2016-17	Subunit	Trivalent	Novartis Vaccines	A/Christchurch/16/2010 NIB-74	A/Hong Kong/4801/2014 NYMC X-263B	-	B/Brisbane/60/2008	Beta-propiolactone	Centrifugation with nonylphenol ethoxylate	Zonal centrifugation
Fluzone 2014-15	Split virus	Quadrivalent	Sanofi Pasteur	A/California/07/2009 X-179A	A/Texas/50/2012 NYMC X-223A	B/Massachusetts/02/2012	B/Brisbane/16/2008	Formaldehyde	Octylphenol ethoxylate (Triton X-100)	A linear sucrose denisty gradient solution using continuous flow centrifuge
Fluzone 2015-16	Split virus	Trivalent	Sanofi Pasteur	A/California/07/2009 X-179A	A/Switzerland/9715293/2013 NIB-88	B/Phuket/3073/2013	-	Formaldehyde	Octylphenol ethoxylate (Triton X-100)	A linear sucrose denisty gradient solution using continuous flow centrifuge

Fluzone 2016-17	Split virus	Quadrivalent	Sanofi Pasteur	A/California/07/2009 X-179A	A/Hong Kong/4801/2014	B/Phuket/3073/2013	B/Brisbane/16/2008	Formaldehyde	Octylphenol ethoxylate (Triton X-100)	A linear sucrose denisty gradient solution using continuous flow centrifuge
Fluzone 2017-18	Split virus	Quadrivalent	Sanofi Pasteur	A/California/07/2009 X-179A	A/Switzerland/9715293/2013 NIB-88	B/Phuket/3073/2013	B/Brisbane/16/2008	Formaldehyde	Octylphenol ethoxylate (Triton X-100)	A linear sucrose denisty gradient solution using continuous flow centrifuge
Fluzone 2018-19	Split virus	Quadrivalent	Sanofi Pasteur	A/Michigan/45/2015 X-275	A/Singapore/INFIMH-16-0019/2016 IVR-86	B/Phuket/3073/2013	B/Maryland/15/2016 BX-69A	Formaldehyde	Octylphenol ethoxylate (Triton X-100)	A linear sucrose denisty gradient solution using continuous flow centrifuge
Flucelvax 2015-16	Subunit	Quadrivalent	Novartis Vaccines	A/Brisbane/10/2010	A/South Australia/55/2014	B/Utah/9/2014	-	Beta-propiolactone	Cetyltrimethy-lammonium bromide	
Flucelvax 2016-17	Subunit	Quadrivalent	Novartis Vaccines	A/Brisbane/10/2010	A/Hong Kong/4801/2014	B/Utah/9/2014	B/Hong Kong/259/2010	Beta-propiolactone	Cetyltrimethy-lammonium bromide	
Flublok 2016-17	Recombinant	Trivalent	Protein Sciences	A/California/07/2009	A/Hong Kong/4801/2014	-	B/Brisbane/60/2008	-	Triton X-100	Column chromatography
Flublok 2016-17	Recombinant	Trivalent	Protein Sciences	A/California/07/2009	A/Hong Kong/4801/2014	-	B/Brisbane/60/2008	-	Triton X-100	Column chromatography

Supplemental Table I. Summary of the vaccines used to study the influenza vaccine composition and protein interactions.

Target Protein	Antibody ID	Antibody Clone	Specificity	Species	Clonality	Source	Use in Study
H1	4F8	4F8	A/California/04/2009 (H1N1) pdm09	Mouse	Mono	BEI Resource<br>Cat no. NR-42021	WB
H1	3E05	019 3E05	A/California/04/2009 (H1N1) Head Domain	Human	Mono	P. Wilson	IP
H1	5C01	051-051310-5C01	A/California/04/2009 (H1N1) Stalk Domain; A/Brisbane/2007 (H1N1)	Human	Mono	P. Wilson	IP
HA	PA5-34929	34929	C-ter of IAV HA A/WSN/1933 (H1N1)	Rabbit	Poly	Thermo Fisher<br>Cat no. PA5-34929	WB
H3	MM05	MM05	H3/H7	Mouse	Mono	SinoBiologicals<br>Cat no. 11056-MM03	WB
H3	MM03	MM03	H3/H7	Mouse	Mono	SinoBiologicals<br>Cat no. 11056-MM05	WB
N1	4A5	4A5	Broadly binds N1<br>(Wohlbold et al 2015)	Mouse	Mono	F. Krammer	WB
N1	21304	21304	C-ter of IAV NA	Rabbit	Poly	Abcam<br>Cat no. ab21304	WB
NP	HB65	HB65	Reactive against A and B strains	Mouse	Mono	J. Yewdell	WB
M1 - A	20910	20910	M1 (influenza A, Philippines H3N2)	Goat	Poly	Abcam<br>Cat no. ab20910	WB
M1 - B	GTX128536	753-05M	B/Taiwan/753/2005 (Yamagata)	Rabbit	Poly	GeneTex	WB
HA-B	B18	B18	Influenza B HA Yamagata and Victoria lineages	Mouse	Mono	Abcam<br>Cat no. ab82604	WB
HA-B	PA5-34975	B70555	B/Taiwan/70555/2005 (Victoria)	Rabbit	Poly	Invitrogen<br>Cat no. PA5-34975	WB
Negative Control	UCD1114<br>TG2-2D04	TG2-2D04	Transglutaminase-2	Human	Mono	P. Wilson	IP

Supplemental Table II. Summary of the antibodies used to study the influenza vaccine composition and protein interactions.

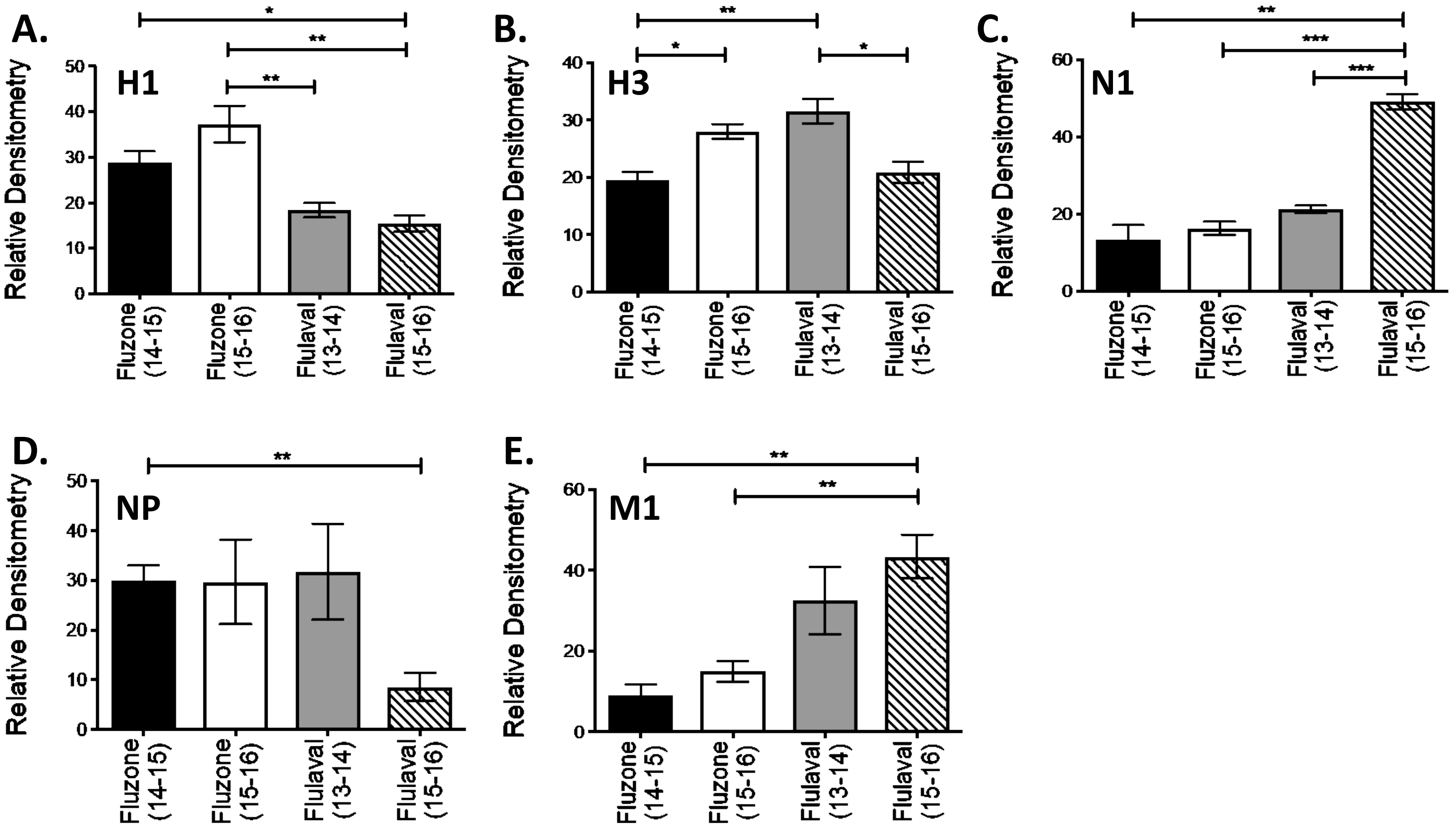

Supplemental Figure 1. Densitometry analysis of Fluzone and FluLaval vaccines for viral protein composition. Each panel illustrates the average relative densitometry for the indicated viral protein (H1 (Panel A), H3 (Panel B), N1 (Panel C), NP (Panel D) and M1 (Panel E)) for each of the four vaccines analyzed in Figure 1 (Fluzone 14-15 (black bar), Fluzone 15-16 (white), FluLaval 13-14 (grey) and FluLaval 15-16 (hatched). In order to normalize within each experiment, for each individual assay, the bands identified by chemiluminescence were scored by the total densitometry signal detected among all detected individual proteins within each vaccine using the program ImageJ. These values were summed, the individual values of each protein within each vaccine were then scored as a percentage of the total signal detected, represented as relative densitometry. In this way, the protein detected in each vaccine was assigned as a fraction of the total of the proteins detected. This allowed the reproducibility of the assays to be evaluated and relative abundance of the viral proteins among the vaccines to be assessed. Values for each protein were average from 3-4 independent experiments at the same exposure. Error bars represent the standard error of the mean and statistical relationships were quantified using an ANOVA with multiple comparison correction.

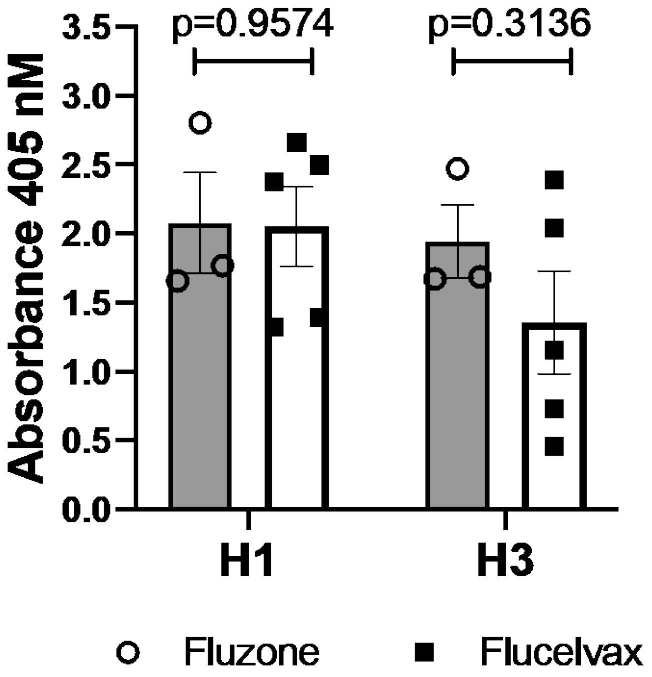

Supplementary Figure 2. HA-specific serum IgG following vaccination with seasonal influenza vaccine. Mice were vaccinated with Fluzone (filled) or Flucelvax (open) and serum antibodies sampled at day 13 were tested for reactivity to recombinant H1 and H3 proteins, as indicated from left to right. IgG antibodies were quantified by ELISA assays and the data are presented at a fixed serum dilution that was in the linear part of the serum titration. HA-B is not shown here because the valency of the vaccines tested were different, with Flucelvax supplied as a trivalent vaccine and Fluzone supplied as a quadrivalent vaccine. Shown is the average of 3-5 individual animals, with individuals indicated by individual points. Error bars represent the standard error of the mean and the student t test was used to calculate p values.

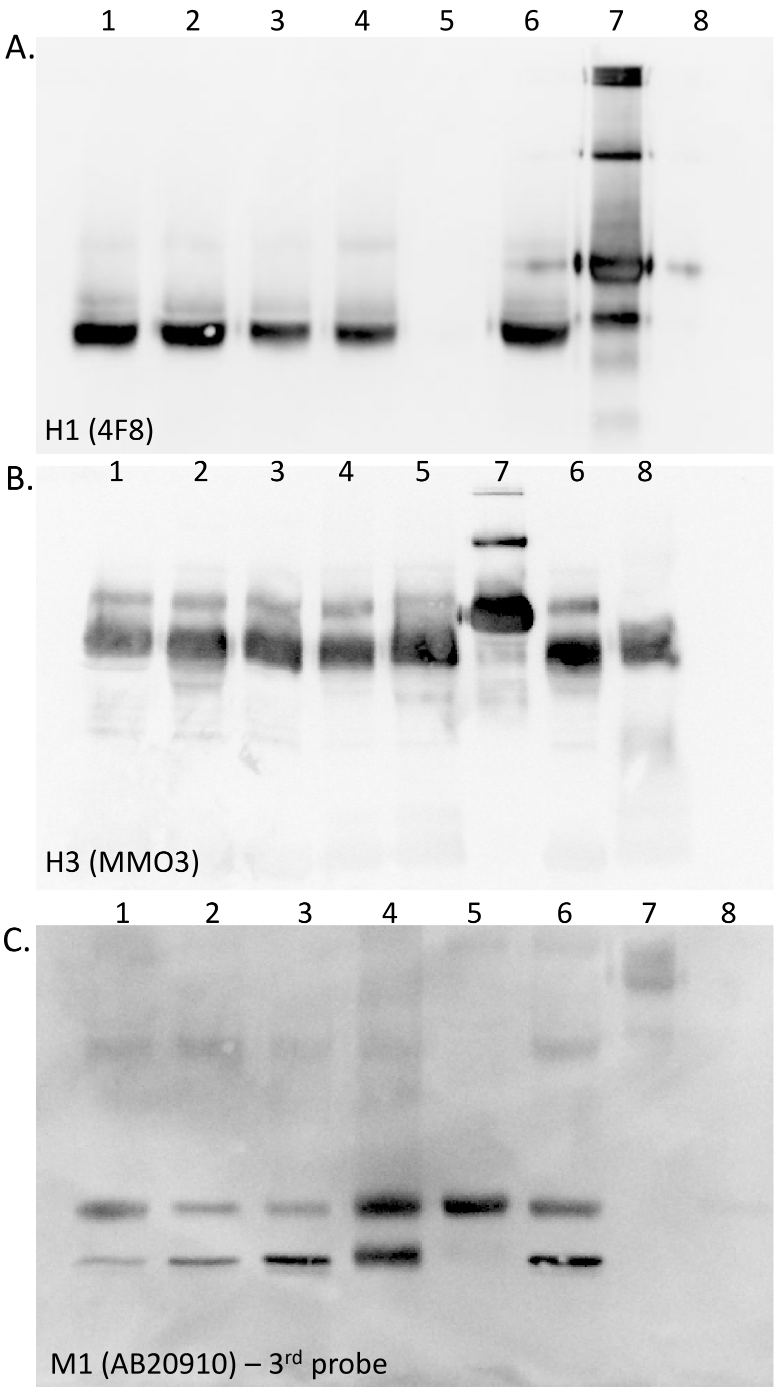

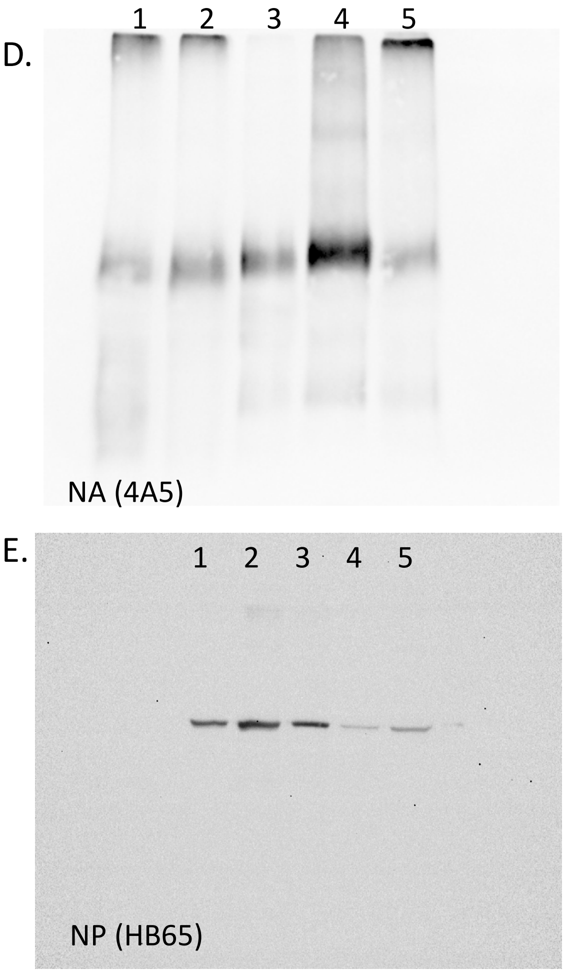

Supplementary Figure 3. Viral protein composition of licensed seasonal influenza vaccines. Shown are the the un-cropped blots presented in Figure 1A. Fluzone 14-15, Fluzone 15-16, FluLaval 13-14, and FluLaval 15-16 are shown in lanes 1-4, respectively. Additional seasonal vaccines included in these analyses but not presented in Figure 1 include, Fluarix 15-16 (lane 5), trivalent Fluzone 15-16 (lane 6), Flublok 15-16 (lane 7) and Flucelvax 15-16 (lane 8). Panel A was probed for H1, panel B was probed for H3, panel C was probed for M1 following probing for H1 (shown in panel A) and N1 (not shown), panel D was probed for N1 and panel E was probed for NP.

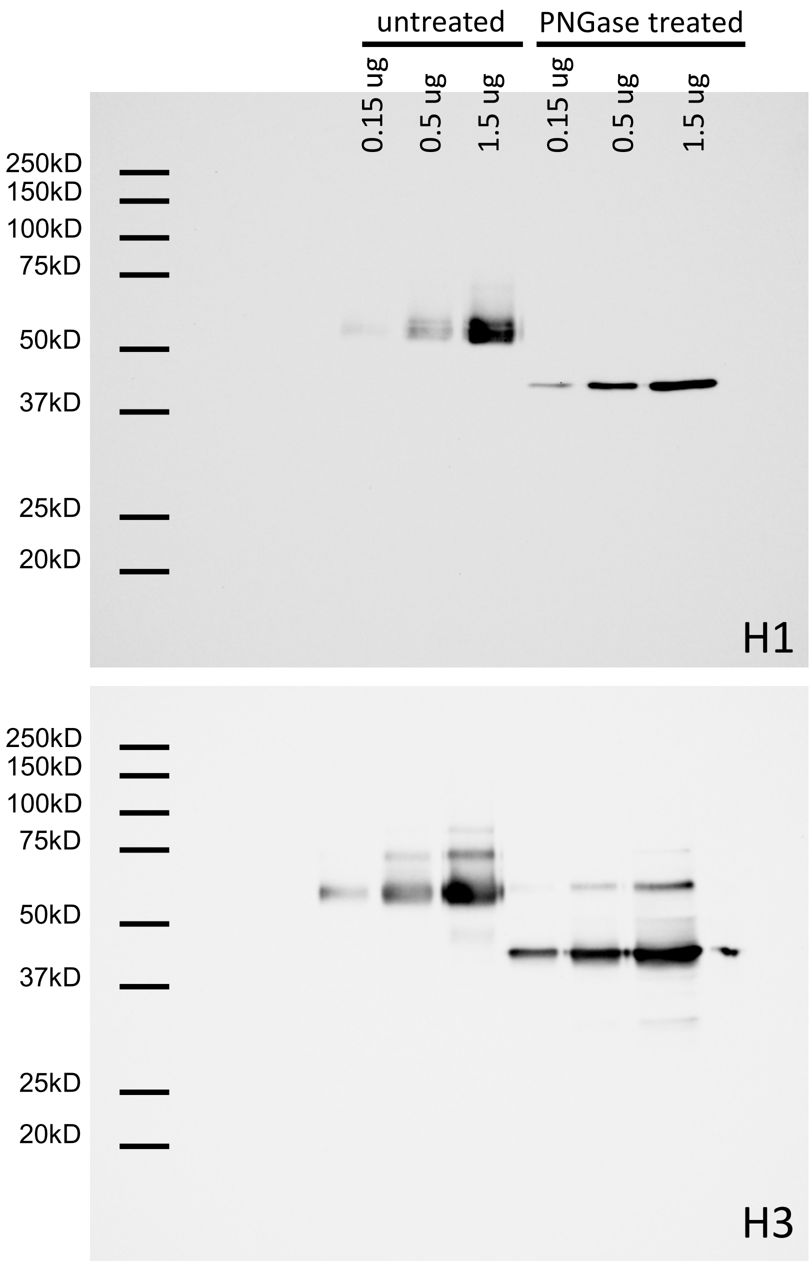

Supplementary Figure 4. Removal of N-glycans on the HA protein reduces the apparent molecular weight and focuses the mobility of HA protein. Shown are the the un-cropped blots presented in Figure 1B. Fluzone proteins in the 2017-18 vaccine were treated with the enzyme PNGase-F to remove any N-linked glycans present on the HA proteins and then Western blots were probed for H1 and H3. Untreated vaccine, applied at the indicated concentrations of HA, are shown on the left and the PNGase-treated vaccines are shown on the right. The top panel shows the Western blot probed for H1 and the bottom panel shows the blot probed for H3. Molecular weight markers are indicated on the left of the Figure.

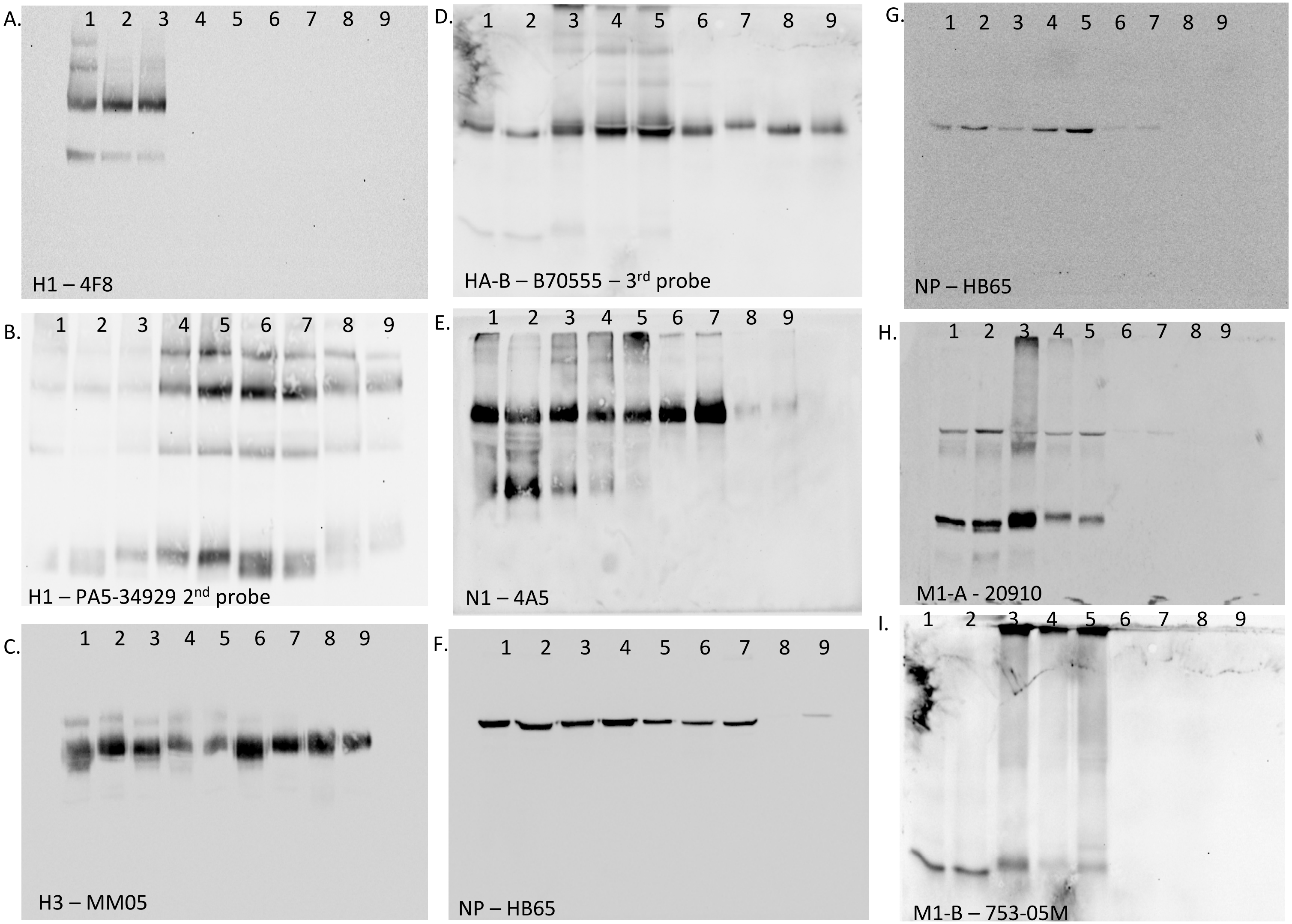

Supplementary Figure 5. Viral protein composition of licensed seasonal influenza vaccines. Shown are the the un-cropped blots presented in Figure 2. Fluzone 15-16 (Lane 1), FluLaval 13-14 (Lane 2), FluLaval 15-16 (Lane 3), Fluarix 15-16 (Lane 4), Fluarix 16-17 (Lane 5), Fluvirin 15-16 (Lane 6), Fluvirin 16-17 (Lane 7), Flucelvax 15-16 (Lane 8) and Flucelvax 16-17 (Lane 9) were surveyed for H1 (panels A and B), H3 (panel C), HA-B (panel D), N1 (panel E), NP (panels F and G), M1-A (panel H) and M1-B (panel I), as indicated in the bottom left corner of each blot. H1 was probed with antibody 4F8 (panel A) followed by PA5-34929 (panel B). The blot shown in panels G, I and D was probed for NP, followed by M1-B and then HA-B, respectively. Panel F shows the primary blot for NP with the lanes as indicated Fluzone 14-15 (Lanes 1 and 2), Fluzone 15-16 (Lanes 3 and 4), FluLaval 13-14 (Lane 5), Flulaval 15-16 (Lane 6), Fluarix 15-16 (Lane 7) and recombinant NP protein 1ug (Lane 8) and 5ug (Lane 9). Panel H shows the blot for M1-A following detection of NP (not shown).

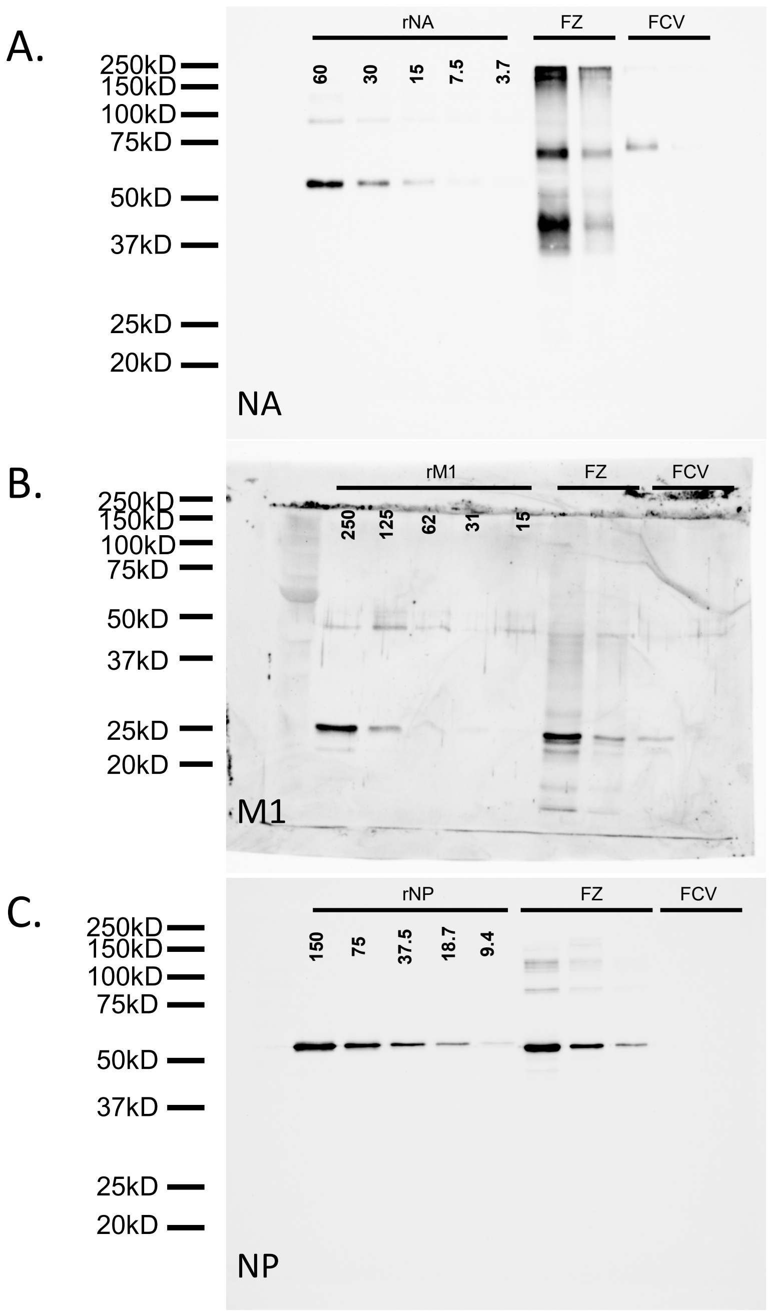

Supplementary Figure 6. Quantification of influenza proteins in seasonal vaccine. Shown are the original blots of data presented in Figure 3. In panel A, recombinant NA protein was applied in two-fold dilutions starting at 60ng. Fluzone (FZ) 14-15 and Flucelvax (FCV) 15-16 were applied at 1.8 or 0.6µg HA, respectively. In panel B, recombinant M1 protein was applied in two-fold dilutions starting at 250ng. Fluzone (FZ) 14-15 and Flucelvax (FCV) 15-16 were applied at 1.8 or 0.6µg HA, respectively. In panel C, recombinant NP protein was applied in two-fold dilutions starting at 150ng, Fluzone (FZ) 14-15 was applied at 0.6µg, 0.2µg and 0.07µg HA, and Flucelvax (FCV) 15-16 was applied at 1.8µg HA, respectively. The mobility of the molecular weight markers is indicated to the left.

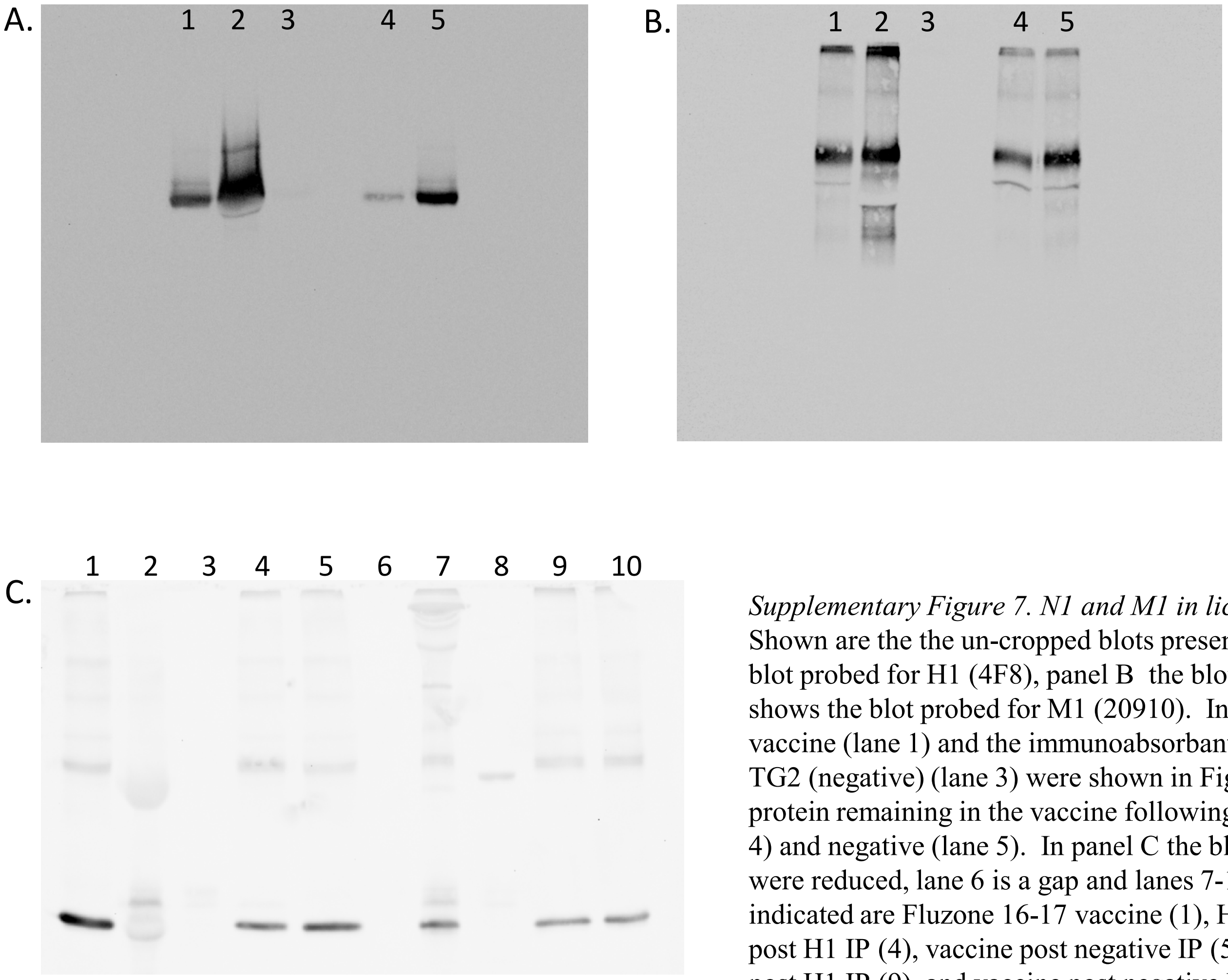

Supplementary Figure 7. N1 and M1 in licensed vaccine co-isolate with H1. Shown are the the un-cropped blots presented in Figure 4. Panel A shows the blot probed for H1 (4F8), panel B the blot probed for N1 (4A5) and panel C shows the blot probed for M1 (20910). In panels A and B Fluzone 16-17 vaccine (lane 1) and the immunoabsorbant with either anti H1 (lane 2) or anti TG2 (negative) (lane 3) were shown in Figure 4. Lanes 4 and 5 indicate the protein remaining in the vaccine following immunoprecipiation with H1 (lane 4) and negative (lane 5). In panel C the blot probed for M1 is shown, lanes 1-5 were reduced, lane 6 is a gap and lanes 7-10 were non-reduced. The lanes as indicated are Fluzone 16-17 vaccine (1), H1 IP (2), Negative IP (3), vaccine post H1 IP (4), vaccine post negative IP (5), H1 IP (7), Negative IP (8), vaccine post H1 IP (9), and vaccine post negative IP (10). Shown in Figure 4 are lanes 1, 6 and 7.

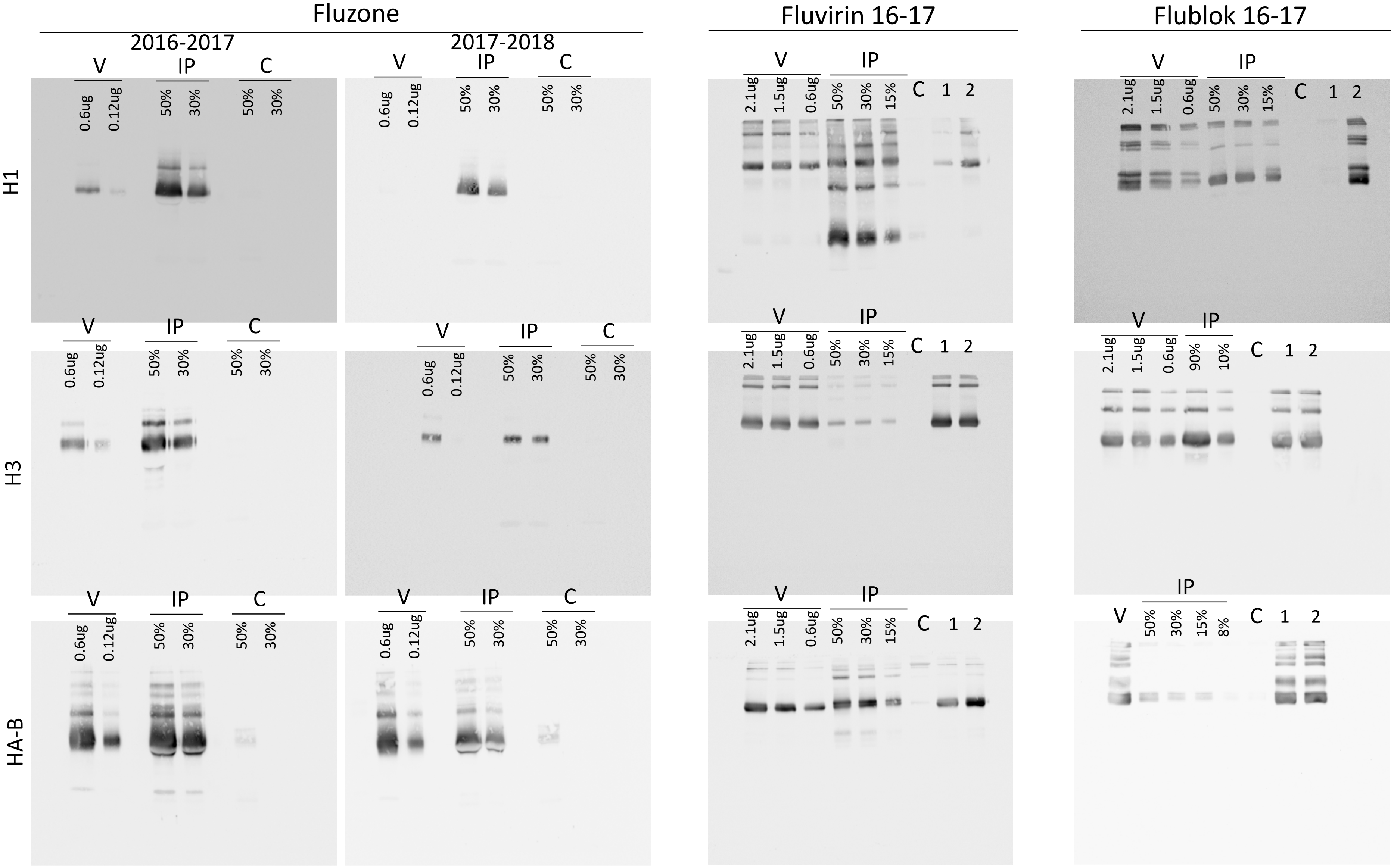

Supplementary Figure 8. Heterologous hemagglutinin proteins co-isolate with H1 in influenza vaccines. Shown are the the un-cropped blots presented in Figure 5. Vaccine (V) was applied at varying quantities of HA as indicated, H1 IPs (IP) were applied at varying quantities of the elution volume as indicated by the percentages, the negative control is indicated by “C” and lanes 1 and 2 indicate the vaccine post H1 IP or control IP, respectively. The probing antibody is indicated to the left and the vaccine is indicated above the panels.

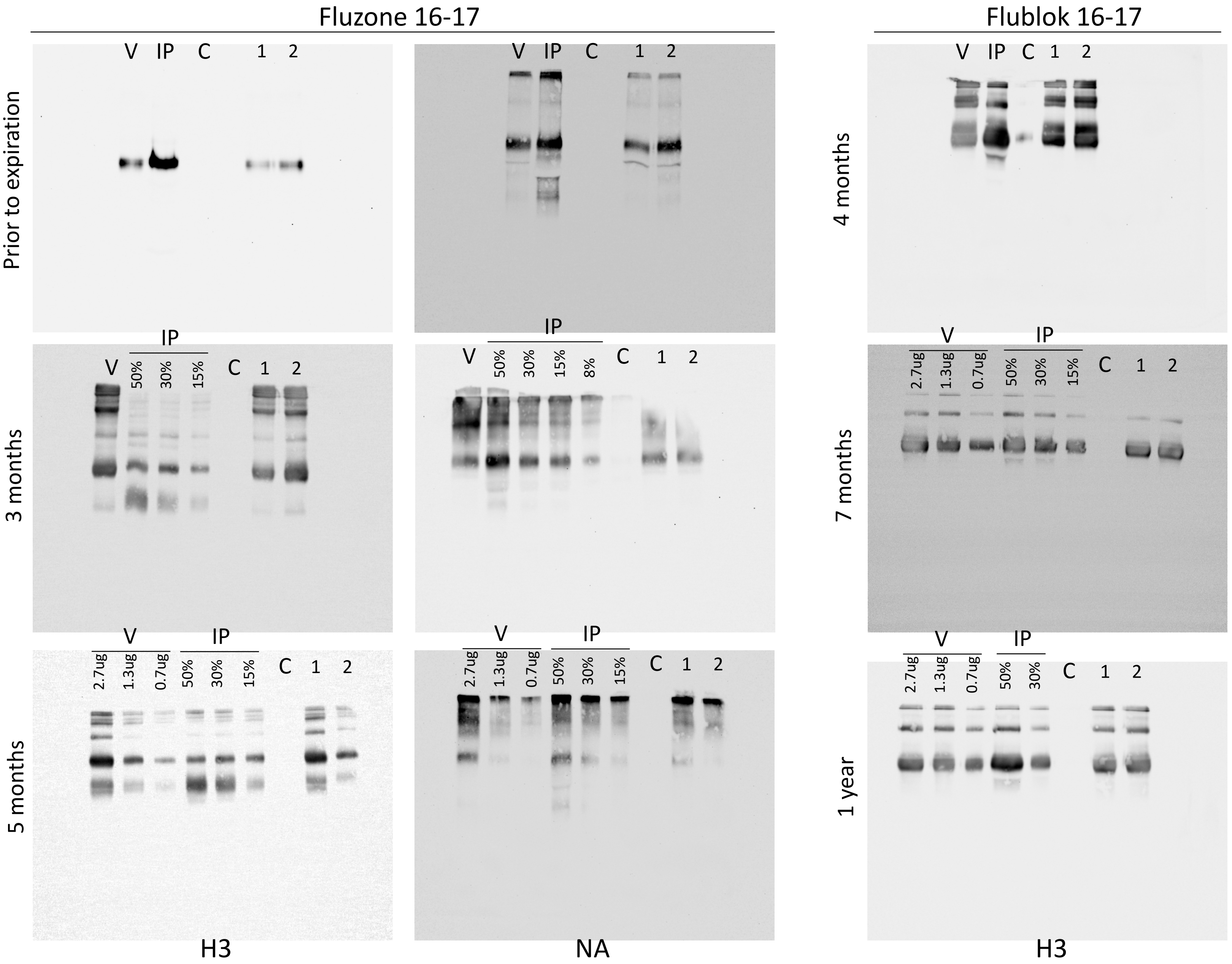

Supplementary Figure 9. Interaction of heterologous hemagglutinin proteins in influenza vaccines are unchanged over time. Shown are the un-cropped blots presented in Figure 6. Vaccine (V) was applied at varying quantities of HA as indicated, H1 IPs (IP) were applied at varying quantities of the elution volume as indicated by the percentages, the negative control is indicated by "C" and lanes 1 and 2 indicate the vaccine post H1 IP or control IP, respectively. The timeframe of the IP post vaccine expiration is indicated to the left of the panels, the vaccine is indicated above the panels and the probing antibody is indicated below the panels.

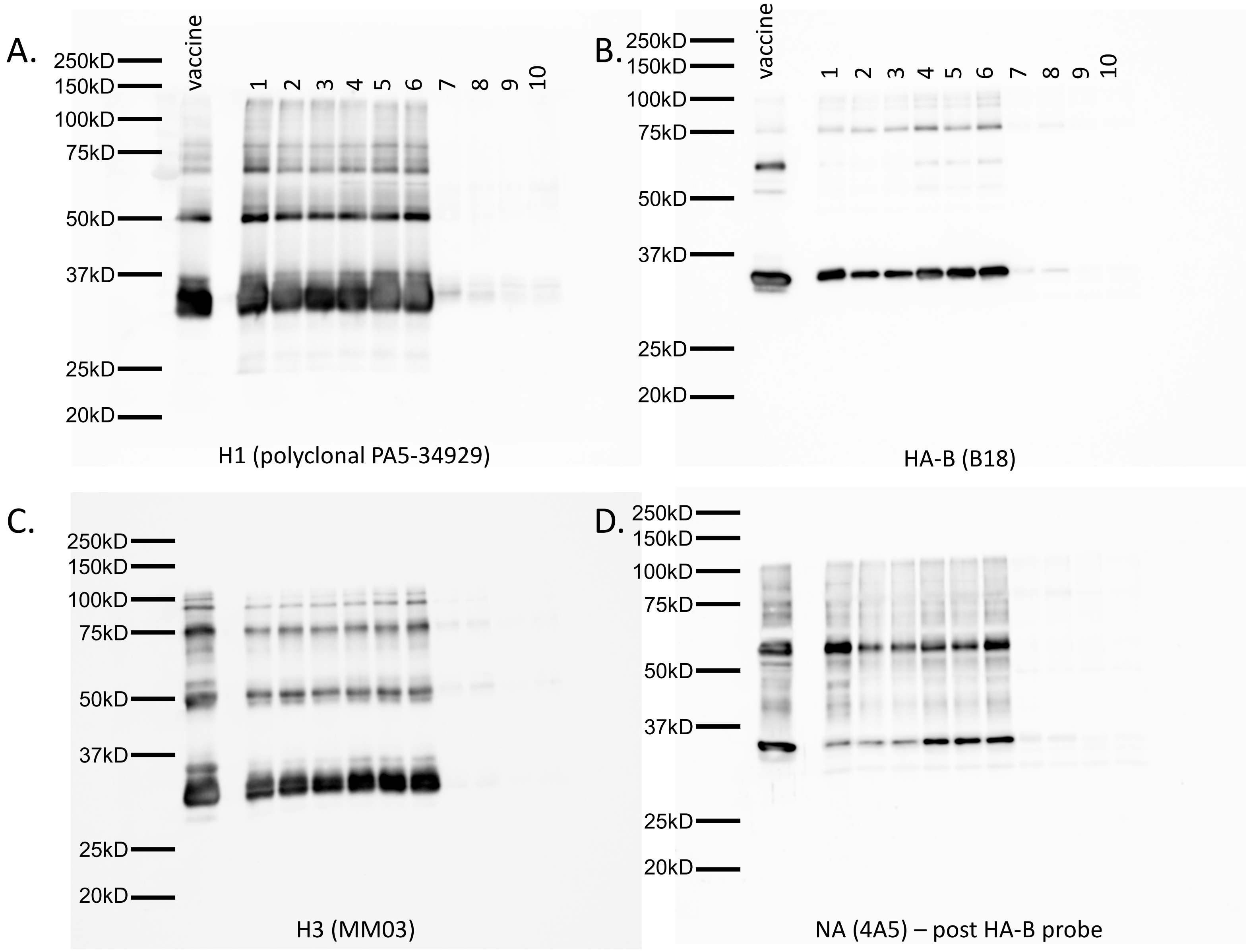

Supplementary Figure 10. Protein-protein interactions in the seasonal influenza vaccine persist at 37°C. Original, un-cropped blots are shown for the data presented in Figure 7. Panel A is the image of the blot probed for H1, Panel B was probed for HA-B, panel C was probed for H3 and panel D was probed for NA following detection of HA-B. Molecular weight markers are indicated to the left of each panel and lanes indicated as 1-10 are as follows: Lanes 1-6 are H1 IPs and Lanes 7-10 are Control IPs. Vaccine was incubated 4°C (lanes 1, 4, 7 and 9) or incubated at 37°C for 2 hr. (lanes 2, 5, 8 and 10) or 4 hr. (lanes 3 and 6) with BSA (lanes 4-6 and 9-10) or without BSA (lanes 1-3 and 7-8). Vaccine (1μg) was applied to the gel as a control in the first lane of each blot as indicated.

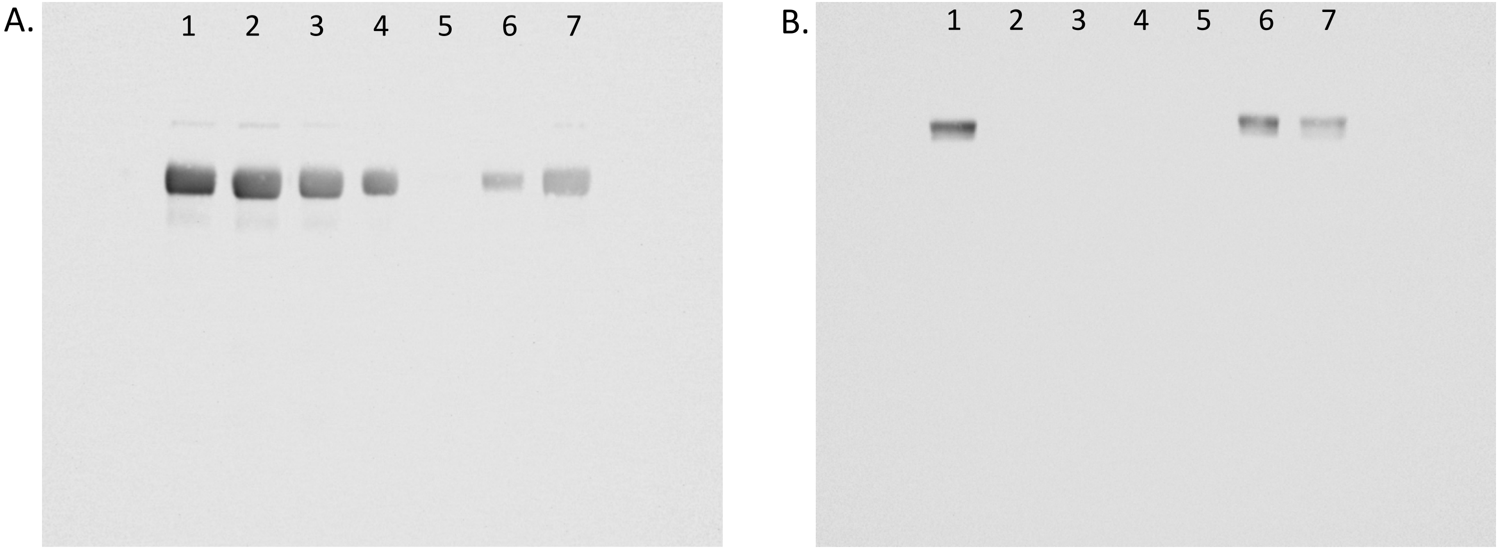

Supplementary Figure 11. The native transmembrane domain is needed for interactions among HA proteins. Shown are the the un-cropped blots presented in Figure 8. Panel A shows the blot probed for H1 (4F8), panel B shows the blot probed for H3 (MM03). In panels A and B, H1 A/California/04/09 (H1) and A/Perth/16/09 (H3) soluble recombinant proteins were combined and applied at 2ug (lane 1) HA, the immunoabsorbant of the combined proteins with either anti-H1 (lanes 2-4) or anti-TG2 (negative) (lane 5) were applied, shown were lanes 1, 2 and 5. Lanes 6 and 7 indicate the protein remaining in the supernatant following immunoprecipiation with H1 (lane 6) and negative (lane 7).
